# GATCL: graph attention network meets contrastive learning for spatial domain identification

**DOI:** 10.1093/bib/bbag043

**Published:** 2026-02-12

**Authors:** Jichong Mu, Yachen Yao, Qiuhao Chen, Jiqiu Sun, Tianyi Zhao

**Affiliations:** School of Computer Science and Technology, Harbin Institute of Technology, Xidazhi St 90, 150000, Harbin, Heilongjiang, China; Zhengzhou Research Institute, Harbin Institute of Technology, Longyuan East Seventh Street, 450000, Zhengzhou, Henan, China; School of Computer Science and Technology, Harbin Institute of Technology, Xidazhi St 90, 150000, Harbin, Heilongjiang, China; School of Computer Science and Technology, Harbin Institute of Technology, Xidazhi St 90, 150000, Harbin, Heilongjiang, China; Zhengzhou Research Institute, Harbin Institute of Technology, Longyuan East Seventh Street, 450000, Zhengzhou, Henan, China; Harbin Institute of Technology Hospital, Xiaowai St, 150000, Harbin, Heilongjiang, China; School of Medicine and Health, Harbin Institute of Technology, Xidazhi St 90, 150000, Harbin, Heilongjiang, China

**Keywords:** spatial domain identification, graph attention network, contrastive learning, spatial multi-omics

## Abstract

Spatial domain identification is an essential task for revealing spatial heterogeneity within tissues, providing insights into disease mechanisms, tissue development, and the cellular microenvironment. In recent years, spatial multi-omics has emerged as the new frontier in spatial domain identification that offers deeper insights into the complex interplay and functional dynamics of heterogeneous cell communities within their native tissue context. Most existing methods rely on static graph structures that treat all neighboring cells uniformly, failing to capture the nuanced cellular interactions within the microenvironment and thus blurring functional boundaries. Furthermore, cross-modal reconstruction performance is often degraded by overfitting to modality-specific noise, which may impair the precise delineation of spatial domains. Therefore, we present GATCL, a novel deep learning framework that integrates a graph attention network with contrastive learning (CL) for robust spatial domain identification. First, GATCL leverages the graph attention mechanism to dynamically assign weights to neighboring spots, adaptively modeling the complex cellular architecture. Second, it implements a cross-modal CL strategy that forces representations from the same spatial location to be similar while pushing those from different locations apart, thereby achieving robust alignment between modalities. Comprehensive experiments across six distinct datasets (spanning transcriptome, proteome, and chromatin) reveal that GATCL is superior to seven representative methods across six key evaluation metrics.

## Introduction

The emergence of single-cell technologies has revolutionized the understanding of cellular heterogeneity and dynamic changes within complex biological systems [1–3]. Following this, spatially resolved omics technologies have emerged as the next major frontier for preserving the native spatial context of cells within tissues [4, 5]. By integrating sequencing data with spatial coordinates, these technologies provide crucial insights into molecular interactions within the tissue’s native microenvironment [6–8]. Spatial transcriptomics technologies are mainly categorized into two types [9–11]: imaging-based technologies (e.g. MERFISH [12], seqFISH+ [13], and osmFISH [14]) and sequencing-based technologies (e.g. 10$\times $ Visium [15], Slide-seq [16], and Stereo-seq [17]). However, due to the inherent limitations of available information in complex tissues, spatial transcriptomics technologies restrict comprehensive analysis. To address this, the field has transitioned to spatial multi-omics, like Stereo-cite-seq [18], SPOTS [19], spatial ATAC–RNA-seq and CUT&Tag-RNA-seq [20].

Spatial domain identification is facilitated by these technologies that aim to identify functional regions within the tissue [21–23]. In recent years, numerous computational methods have been developed for the task. BayesSpace [24] leverages spatial neighborhood information to improve the resolution of spatial transcriptomic data with statistical approach. But it faces restrictions when processing large-scale datasets. Consequently, as a powerful computational tool, deep learning is well-suited for modeling complex biological data [25, 26]. Particularly, graph-based models convert spatial data into a node-edge topology to directly explore spot correlations for domain identification [27]. SpaGCN [28] combines neighboring gene expression to identify spatial domains characterized by consistent expression patterns and histological features. Then DeepST [29] integrates the multi-source data to address the limitation of SpaGCN including failing to model nonlinear interactions and poor handling of multi-source data with dual encoders. However, both SpaGCN and DeepST lack the capability to integrate serial section data. STAGATE [30] builds a cross-section spatial network via an adaptive graph attention autoencoder to enable joint analysis of multiple sections. Considering that STAGATE lacks the capability to remove batch effects, GraphST [5] implicitly corrects batch effects relying on graph-based self-supervised contrastive learning (CL). While most aforementioned methods construct adjacency matrices via predefined similarity metrics or simply fuse multiple data sources additively, STMGCN [31] adopts multiple neighborhood graphs with independent encoders for view-specific representations and an attention mechanism for adaptive fusion. Besides, SpaNCMG [11] constructs a complementary neighborhood graph by fusing local information and global structure information to enhance the spatial transcriptome data.

While spatial transcriptomics has been pivotal in spatial domain identification, the information from multi-omics modalities can help to further enrich the functional characterization. However, the inherent heterogeneity across different omics poses significant computational challenges for integration. Recently, SpatialGlue [32] proposes a graph neural network architecture equipped with a dual-attention mechanism to synergistically integrate spatial multi-omics data. Meanwhile, it leverages cross-modal decoding as an auxiliary task to align features from different modalities. However, conventional GNNs assume uniform neighbor influence, overlooking microenvironmental heterogeneity, which might blur functional boundaries. Furthermore, cross-modal reconstruction may inadvertently focus the model on modality-specific details. Extensive research has shown that augmenting GNNs with attention mechanisms allows capturing more complex relationships by assigning distinct importance weights to neighbors [33]. In addition, CL extracts discriminative embeddings by aligning positive pairs and segregating negatives [34, 35]. Leveraging these established advantages, we propose GATCL: first, its graph attention network preserves clear domain boundaries by assigning higher weights to functionally similar neighbors; second, it implements a CL strategy that maximizes concordance for co-located spots, yielding more discriminative representations for precise spatial domain delineation. Extensive experiments confirm that GATCL consistently surpasses seven representative methods across several datasets and platforms.

## Materials and methods

### Overview of the method

**Figure 1:**
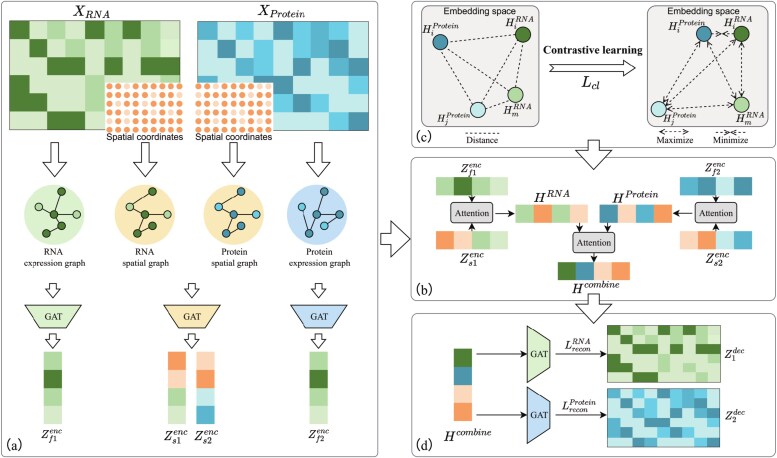
The overall architecture of GATCL.

The architecture of GATCL is visualized in Fig. 1. As shown in Fig. 1a, GATCL constructs two complementary graphs: a spatial graph based on spatial coordinates and a feature similarity graph derived from molecular profiles. Subsequently, these graphs are independently processed by multi-layer graph attention networks to extract latent embeddings. As depicted in Fig. 1b, an intra-modality and a cross-modal attention mechanism fusion module inspired by SpatialGlue [32] adaptively enables joint modeling of spatial multi-omics data. Following that, corresponding to Fig. 1c, GATCL leverages CL to reinforce cross-modal feature consistency by aligning features at spatially corresponding locations and separating those at non-corresponding ones to further enhance consistency. Finally, the model is trained with a joint objective that combines modality-specific reconstruction losses (Fig. 1d) and CL loss (Fig. 1c).

### Graph construction

#### Expression-based graph construction

Given the expression matrix ${X} \in \mathbb{R}^{N \times F}$, where $N$ is the number of spots and $F$ is the feature dimension, we construct a $k$-nearest neighbor (KNN) graph based on feature similarity. The adjacency matrix ${A}_{\mathrm{f}} \in \mathbb{R}^{N \times N}$ is defined as:


(1) \begin{align*}& {A}_{\mathrm{f}}(i, j) = \begin{cases} 1, & \text{if } j \in \mathcal{N}_{k}^{\mathrm{feat}}(i) \\ 0, & \mathrm{otherwise} \end{cases}\end{align*}

where $\mathcal{N}_{k}^{\mathrm{feat}}(i)$ denotes the $k$ most correlated spots to spot $i$. This process is applied independently to the different modalities, resulting in two distinct graphs: ${A}_{\mathrm{f}}^{1}$ and ${A}_{\mathrm{f}}^{2}$.

#### Spatial-based graph construction

To capture the spatial relationships between different spots, we construct a spatial proximity graph. Based on Euclidean distances between the physical coordinates of the spots, the adjacency matrix ${A}_{\mathrm{s}} \in \{0,1\}^{N \times N}$ is defined as:


(2) \begin{align*}& {A}_{\mathrm{s}}(i, j) = \begin{cases} 1, & \text{if } j \in \mathcal{N}_{k}^{\mathrm{spatial}}(i) \\ 0, & \mathrm{otherwise} \end{cases}\end{align*}

Here, $\mathcal{N}_{k}^{\mathrm{spatial}}(i)$ denotes the KNN of node $i$ based on the Euclidean distance:


(3) \begin{align*}& \mathrm{Dist}_{\mathrm{euclid}}({s}_{i}, {s}_{j}) = \|{s}_{i} - {s}_{j}\|_{2}\end{align*}

where $s_{i}$ and $s_{j}$ are the spatial coordinates of spot $i$ and spot $j$, respectively. And the process efficiently identifies nearest neighbors in physical space, also resulting in two graphs: ${A}_{\mathrm{s}}^{1}$ and ${A}_{\mathrm{s}}^{2}$.

### Graph attention network

To encode the graph-structured data, we employ an $L$-layer GAT encoder. By dynamically weighting neighbors, the GAT focuses on the more important nodes for effective representation learning. Besides, this design is also applied to the decoder, differing in its inputs and outputs. In short, the process can be described as follows:


(4) \begin{align*} & Z^{\mathrm{enc}}_{f1} = {\mathrm{GATConv}}\Big(X^{\mathrm{RNA}},A_{f}^{1}\Big) \end{align*}

(5) \begin{align*} & Z^{\mathrm{enc}}_{s1} = {\mathrm{GATConv}}\Big(X^{\mathrm{RNA}},A_{s}^{1}\Big) \end{align*}

(6) \begin{align*} & Z^{\mathrm{enc}}_{f2} = {\mathrm{GATConv}}\Big(X^{\mathrm{Protein}},A_{f}^{2}\Big) \end{align*}

(7) \begin{align*} & Z^{\mathrm{enc}}_{s2} = {\mathrm{GATConv}}\Big(X^{\mathrm{Protein}},A_{s}^{2}\Big) \end{align*}

where $X^{\mathrm{RNA}}$ and $X^{\mathrm{Protein}}$ are the expression matrices of the transcriptome and proteome, respectively. Here, GATConv represents the multi-layer graph attention network encoder which is formally defined as follows. let $\mathcal{G} = (\mathcal{V}, \mathcal{E})$ denote a graph with $N = |\mathcal{V}|$ nodes and edge set $\mathcal{E}$. The initial node features are represented as ${X}^{(0)} \in \mathbb{R}^{N \times F}$, where $F$ is the feature dimension. The $l$th layer ($l = 1, \dots , L$) computes the hidden representation via:


(8) \begin{align*}& {H}^{(l)}_{i} = \begin{cases} \displaystyle\big\|_{k=1}^{K} \sum_{j \in \mathcal{N}(i)} \alpha_{ij}^{(k)} {W}^{(k)} \left( {X}^{(l-1)} \right)_{j}, & \text{if multi-head} \\ \displaystyle\sum_{j \in \mathcal{N}(i)} \alpha_{ij} {W} \left({X}^{(l-1)} \right)_{j}, & \text{if single-head} \end{cases}\end{align*}

where $K$ is the number of attention heads and where ∥ indicates concatenation across $K$ attention heads. The attention coefficients are computed as:


(9) \begin{align*}& \alpha_{ij}^{(k)} = \frac{ \exp\left( \mathrm{LeakyReLU} \left( {a}^{(k)\top} \left[{W}^{(k)}{X}_{i}^{(l-1)} \| {W}^{(k)}{X}_{j}^{(l-1)} \right] \right) \right) }{ \sum_{j \in \mathcal{N}(i)} \exp\left( \mathrm{LeakyReLU} \left( {a}^{(k)\top} \left[{W}^{(k)}{X}_{i}^{(l-1)} \| {W}^{(k)}{X}_{j}^{(l-1)} \right] \right) \right) }\end{align*}

The output of each layer is then updated as:


(10) \begin{align*}& {X}^{(l)} = \mathrm{Dropout} \left( \mathrm{LayerNorm} \left( \sigma({H}^{(l)}) \right) \right) + \mathrm{Proj}^{(l)}({X}^{(l-1)})\end{align*}

Here, $\sigma (\cdot )$ denotes an activation function. The residual projection $\mathrm{Proj}^{(l)}$ is defined as:


\begin{align*} & \mathrm{Proj}^{(l)}({X}^{(l-1)}) = \begin{cases} {X}^{(l-1)}{W}_{\mathrm{res}}^{(l)}, & \text{if } \mathrm{dim}({X}^{(l-1)}) \ne \mathrm{dim}({H}^{(l)}) \\{X}^{(l-1)}, & \mathrm{otherwise} \end{cases} \end{align*}

After $L$ such layers, the encoder outputs ${Z}_{\mathrm{enc}} ={X}^{(L)}$, including transcriptome feature embedding $Z^{\mathrm{enc}}_{f1}$, transcriptome spatial embedding $Z^{\mathrm{enc}}_{s1}$, proteome feature embedding $Z^{\mathrm{enc}}_{f2}$, proteome spatial embedding $Z^{\mathrm{enc}}_{s2}$.

### Modality-aware attention fusion

To adaptively fuse representations from different modalities, we employ a self-learned attention mechanism from SpatialGlue that computes weighted combinations of input embeddings based on their importance:


(11) \begin{align*} & H^{\mathrm{RNA}} = {\mathrm{Attention}}\Big(Z^{\mathrm{enc}}_{f1},Z^{\mathrm{enc}}_{s1}\Big) \end{align*}

(12) \begin{align*} & H^{\mathrm{Protein}} = {\mathrm{Attention}}\Big(Z^{\mathrm{enc}}_{f2}, Z^{\mathrm{enc}}_{s2}\Big) \end{align*}

(13) \begin{align*} & H^{\mathrm{combine}} = {\mathrm{Attention}}\Big(H^{\mathrm{RNA}}, H^{\mathrm{Protein}}\Big) \end{align*}

In detail, given $m$ modality-specific embeddings ${Z_{1}}$, ${Z_{2}}$...${Z_{m}}$, we first concatenate them into a unified tensor:


(14) \begin{align*}& {Z} = \left[{Z_{1}}, {Z_{2}},..., {Z_{m}} \right]\end{align*}

Then apply a two-layer feed-forward attention network to compute attention weights $\alpha _{i}$ over the modality inputs:


(15) \begin{align*} & {v}_{i} = \tanh({Z} {W}_\omega) \end{align*}

(16) \begin{align*} & \alpha_{i} = \frac{\exp({v}_{i}^\top{u}_\omega)}{\sum_{j=1}^{m} \exp({v}_{j}^\top{u}_\omega)} \end{align*}

The attention scores $\alpha _{i}$ reflect the relative importance of each modality. The final fused embedding is computed as the weighted sum:


(17) \begin{align*}& {Z}^{\prime} = \sum_{i=1}^{m} \alpha_{i} {Z}_{i}\end{align*}

### Cross-modality contrastive learning

To align the representations of spatial transcriptomics and proteomics at each spatial location (spot), we adopt a CL framework. We define the positive sample pair as transcriptomic and proteomic data originating from the same location. In contrast, the negative sample pair is constituted by any two omics datasets from different locations. Given $\{{H}_{i}^{\mathrm{RNA}}\}_{i=1}^{N}$, $\{{H}_{i}^{\mathrm{Protein}}\}_{i=1}^{N}$, where $N$ represents the number of spots, the goal is to bring the matched pairs from the same location closer while pushing unmatched pairs apart in the latent space.

Firstly, the representations are L2-normalized:


(18) \begin{align*}& \tilde{{H}}_{i}^{\mathrm{RNA}} = \frac{{H}_{i}^{\mathrm{RNA}}}{\| {H}_{i}^{\mathrm{RNA}} \|_{2}}, \quad \tilde{{H}}_{i}^{\mathrm{Protein}} = \frac{{H}_{i}^{\mathrm{Protein}}}{\| {H}_{i}^{\mathrm{Protein}} \|_{2}}\end{align*}

The similarity between positive pairs (same spot) is computed via dot product:


(19) \begin{align*}& s_{i}^{+} = \left\langle \tilde{{H}}_{i}^{\mathrm{RNA}}, \tilde{{H}}_{i}^{\mathrm{Protein}} \right\rangle\end{align*}

Then the sum of similarities between negative sample pairs $s_{i,j}^{-}$ can be represented as:


(20) \begin{align*} \begin{split} s_{i,j}^{-} ={}& \left\langle \tilde{{H}}_{i}^{\mathrm{RNA}}, \tilde{{H}}_{j}^{\mathrm{Protein}} \right\rangle + \left\langle \tilde{{H}}_{i}^{\mathrm{Protein}}, \tilde{{H}}_{j}^{\mathrm{RNA}} \right\rangle \\ & + \left\langle \tilde{{H}}_{i}^{\mathrm{Protein}}, \tilde{{H}}_{j}^{\mathrm{Protein}} \right\rangle + \left\langle \tilde{{H}}_{i}^{\mathrm{RNA}}, \tilde{{H}}_{j}^{\mathrm{RNA}} \right\rangle, \\ & \quad j \in \mathcal{N}_{\mathrm{neg}}(i), j \neq i \end{split}\end{align*}

The contrastive loss is defined as:


(21) \begin{align*}& \mathcal{L}_{\mathrm{cl}} = - \frac{1}{N} \sum_{i=1}^{N} \log \frac{\exp(s_{i}^{+} / \tau)}{\exp(s_{i}^{+} / \tau) + \sum_{j \in \mathcal{N}_{\mathrm{neg}}(i)} \exp(s_{i,j}^{-} / \tau)}\end{align*}

where $\tau $ is a learnable temperature parameter that is dynamically annealed during training:


(22) \begin{align*}& \tau \leftarrow \tau \cdot \lambda \quad \text{(every 100 steps)}\end{align*}

where annealing factor $\lambda < 1$. This encourages the encoder to produce modality-invariant representations for each spatial spot while maintaining inter-spot discriminability.

### Training objective

To jointly model spatial transcriptome and proteome, we design a multi-task loss that combines both modality-specific reconstruction and cross-modality alignment via CL. The overall objective encourages the model to preserve the original omics information while enforcing consistency between modalities in a shared embedding space. The decoder adopts a symmetric structure similar to the encoder, decoding $H^{\mathrm{combine}}$ separately back into the original transcriptomic and proteomic data to obtain the reconstructed representations $Z^{\mathrm{dec}}_{1}$ and $Z^{\mathrm{dec}}_{2}$. The reconstruction loss for transcriptomics and proteomics is computed as


(23) \begin{align*} & \mathcal{L}_{\mathrm{recon}}^{\mathrm{RNA}} = \frac{1}{N} \sum_{i=1}^{N} \left\| {Z}^{\mathrm{dec}}_{1,i} - {{X_{i}}}^{\mathrm{RNA}} \right\|_{F}^{2} \end{align*}

(24) \begin{align*} & \mathcal{L}_{\mathrm{recon}}^{\mathrm{Protein}} = \frac{1}{N} \sum_{i=1}^{N} \left\| {Z}^{\mathrm{dec}}_{2,i} - {{X_{i}}}^{\mathrm{Protein}} \right\|_{F}^{2} \end{align*}

The total loss can be defined as


(25) \begin{align*}& \mathcal{L}_{\mathrm{total}} = \lambda_{1} \cdot \mathcal{L}_{\mathrm{recon}}^{\mathrm{RNA}} + \lambda_{2} \cdot \mathcal{L}_{\mathrm{recon}}^{\mathrm{Protein}} + \lambda_{3} \cdot \mathcal{L}_{\mathrm{cl}}\end{align*}

where $ \lambda _{1} $, $ \lambda _{2} $ and $ \lambda _{3} $ are hyperparameters.

## Result

### Application to human lymph node A1 dataset

**Figure 2:**
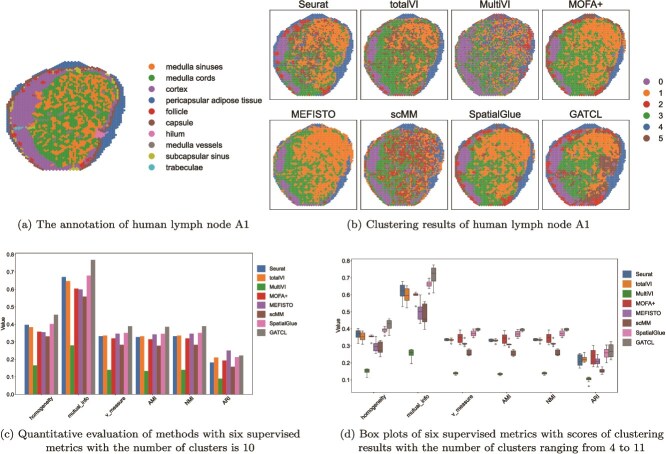
GATCL identifies spatial domains in human lymph node A1.

We first apply GATCL to the human lymph node A1 dataset [32] downloaded from https://zenodo.org/records/10362607, and compare it with several mainstream methods, including Seurat [36], totalVI [37], MultiVI [38], MOFA+ [39], MEFISTO [40], scMM [41], and SpatialGlue [32]. The ground truth is shown in Fig. 2a that is annotated by experts from SpatialGlue [32]. Referring to the ground truth, GATCL exhibits stronger structural consistency and clearer spatial boundaries compared with other methods as presented in Fig. 2b. In detail, GATCL demonstrates more accurate boundaries over others in identifying the pericapsular adipose tissue and the cortex.

In addition, to more intuitively compare the performance of different methods, we select six supervised metrics including homogeneity, mutual information, V-measure, AMI, NMI, and ARI to quantify the results, where higher values indicate better performance. Figure 2c presents the performance comparison when the number of clusters is set to 10, matching the number of ground truth categories referring to Fig. 2a. The results indicate that GATCL either outperforms or performs comparably to the competing methods. For instance, GATCL’s mutual_info score is 0.490 points higher than that of MultiVI, and its homogeneity score is 0.290 points higher than MultiVI’s. Besides, for mutual_info, GATCL achieves a score 0.090 higher than the strongest competitor SpatialGlue. In order to rigorously assess the clustering performance, we conduct a comprehensive comparison, varying the number of clusters from 4 to 11. From the results summarized in Fig. 2d, it is evident that GATCL demonstrates superior performance. Specifically, on all six metrics, GATCL consistently achieves the highest median scores, significantly outperforming all other baseline models. These findings collectively validate the effectiveness and robustness of the GATCL framework for identifying accurate spatial domains.

### Application to human lymph node D1 dataset

**Figure 3:**
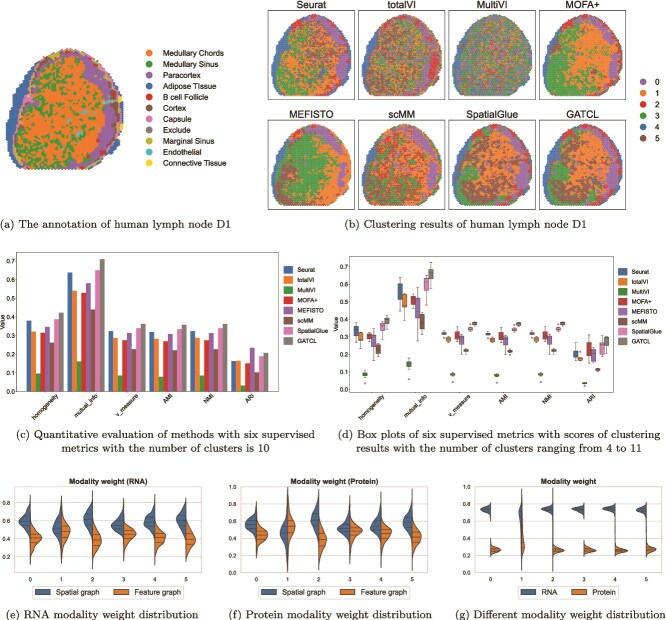
GATCL identifies spatial domains in human lymph node D1.

We replicate the analysis on another human lymph node dataset that could be downloaded from https://zenodo.org/records/10362607. The ground truth labels shown in Fig. 3a are annotated by experts from SpatialGlue [32]. As shown in Fig. 3b, GATCL and SpatialGlue show high concordance with key anatomical regions. In contrast, many mainstream methods (such as Seurat and totalVI) can only resolve indistinct macroscopic structures while ScMM and MultiVI appear severely fragmented.

Then, to ensure the results are not tissue-specific, we compare the supervised metrics (homogeneity, mutual_info, V_measure, AMI, NMI, and ARI) when the number of clusters is 10, identical to the analysis of A1. As is evident from Fig. 3, GATCL continues to show excellent performance. Compared with SpatialGlue, GATCL increases 0.0597 in mutual_info and 0.0356 in homogeneity. Furthermore, GATCL substantially outperforms the Seurat baseline, achieving an ARI score 0.0436 points higher and a mutual_info score 0.0724 points higher. Although its ARI value is slightly lower than that of the MEFISTO method, all other metrics are superior to those of the other methods, including SpatialGlue which has the best performance among all compared methods in spatial domain identification.

Next, we compare GATCL against seven other methods with the number of cluster ranging from 4 to 11 to quantitatively evaluate the robustness and stability of GATCL. The results shown in Fig. 3d demonstrate the superiority of GATCL as it consistently achieves the highest median values across all metrics. These quantitative results provide robust support for the qualitative observations, confirming that GATCL can more accurately and reliably identify biological domains.

To further investigate the modality-specific contributions within GATCL, we visualize the learned modality weights. As shown in Fig. 3e–f, the higher importance weighting of spatial information over feature information for both RNA and protein almost across clusters suggests that spatial proximity offers valuable complementary insights for representation learning. Besides, a significantly higher weight is assigned to RNA than protein as illustrated in Fig. 3g. It indicates that the model primarily relies on information from RNA for its analysis, treating protein information as secondary and supplementary.

### Application to mouse spleen dataset

**Figure 4:**
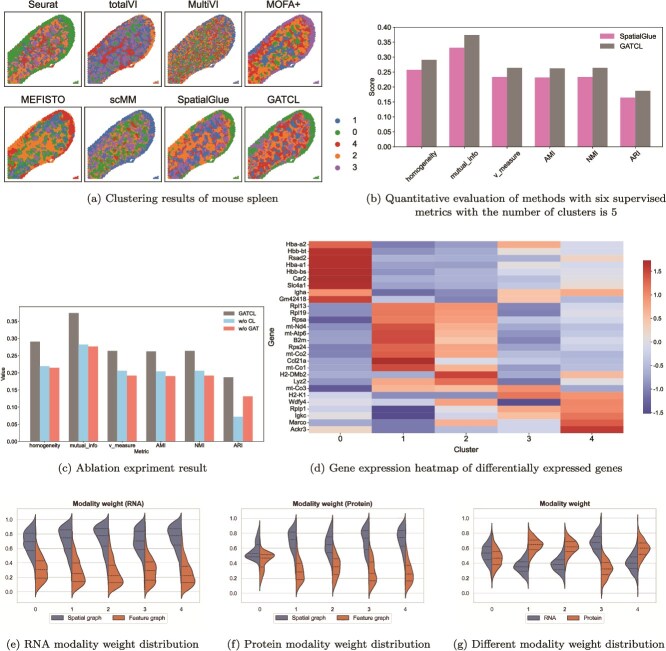
GATCL identifies spatial domains in mouse spleen.

We next extend the testing to a mouse spleen dataset [19, 32] that is downloaded from https://www.ncbi.nlm.nih.gov/geo/query/acc.cgi?acc=GSE198353. Firstly, We visualize the results of each method in Fig. 4a. From Fig. 4a, GATCL and SpatialGlue demonstrate superior performance than others, yielding relatively clear clustering results with distinct boundaries. In stark contrast, most competing methods (such as ScMM, totalVI, and MultiVI) yield results characterized by noise and blurred boundaries. For a more targeted quantitative assessment, we benchmark GATCL against the well-performing SpatialGlue with evaluation metrics (homogeneity, mutual_info, v_measure, AMI, NMI, and ARI). As illustrated in Fig. 4b, GATCL’s performance is consistently superior to SpatialGlue across all metrics, underscoring the higher alignment between its predicted clusters and the ground-truth biological domains. Together, these quantitative findings and the spatial visualizations conclude that GATCL enables more accurate and interpretable spatial domain identification than the compared methods.

Secondly, an ablation experiment confirms the necessity of both the GAT and CL modules (Fig. 4c). Indeed, the full GATCL model consistently outperforms variants lacking either component. Removing the CL module (w/o CL), for instance, significantly degraded performance (e.g. lower mutual-info score), demonstrating its importance for feature representation. Likewise, removing the GAT (w/o GAT) yields the lowest ARI score, highlighting its critical role in capturing spatial proximity and cell-to-cell relationships. These findings confirm that both components are essential for superior spatial domain identification.

Thirdly, we perform differential gene expression analysis across the five predicted domains to evaluate the functional relevance of spatial clusters identified by GATCL. The resulting heatmap shown in Fig. 4d reveals distinct transcriptional profiles that correlate well with the spatial context and known immune cell distributions in the spleen. In Cluster 0, the high expression of Hbb-bs, Hba-a1/a2, Slc4a1, and Gm42418 are markers for erythrocytes to the red pulp macrophages region of the mouse spleen. Cluster 1 is characterized by upregulation of mitochondrial-related genes (e.g. mt-Co1, mt-Co2, and mt-Nd4) and ribosomal protein genes (e.g. Rpl13 and Rpl19), which is a hallmark of highly activated immune cells. Cluster 2 highly expresses H2-DMb2, B2m, etc., all of which are genes related to major histocompatibility complex (MHC) molecules and are rich in antigen-presenting cell regions, representing marginal regions.

Finally, we analyze the modality weights in Fig. 4e–g. From Fig. 4e and f, the model consistently assigns greater importance to cellular spatial relationships for both RNA and protein, indicating a high dependency on the local microenvironment for the predictions. Furthermore, as shown in Fig. 4g, the fused modality weights reveal a shift from RNA-dominant to protein-dominant contributions in certain regions, underscoring the spatially heterogeneous nature of multimodal information.

### Application to mouse thymus dataset

**Figure 5:**
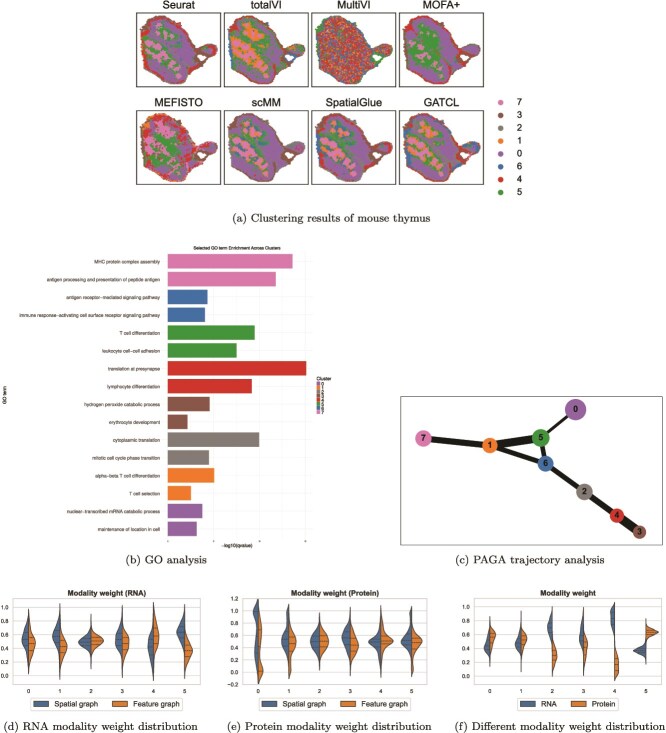
GATCL identifies spatial domains in mouse thymus.

We conduct experiments on the mouse thymus dataset [32] downloaded from https://zenodo.org/records/10362607 that contains highly structured and functionally diverse immune microenvironments. Owing to the lack of established annotations, a quantitative evaluation is infeasible. So, the performance evaluation is instead based on the biological relevance of the identified clustered regions. To assess GATCL’s performance in spatial domain identification, firstly, we benchmark it against seven mainstream methods in Fig. 5a. The results indicate that GATCL, similar to top-performing methods like Seurat and SpatialGlue, successfully identifies spatially coherent domains. These domains clearly mirror the thymus’s core anatomy: an outer cortex (Cluster 0) enveloping a central medulla comprised of various colored clusters (Cluster 5, Cluster 1 and Cluster 7). Conversely, totalVI and MEFISTO yield more fragmented results, and MultiVI fails to resolve any meaningful spatial organization. Overall, GATCL shows better performance stability when partitioning biologically complex tissues.

Secondly, GO analysis further validates the identified spatial domains (Fig. 5), revealing a strong correspondence between their spatial positioning and functional enrichments, which accurately recapitulates the known thymic microarchitecture. Specifically, peripheral and capsular regions (Clusters 0, 1) were enriched for metabolic pathways. The cluster at the corticomedullary junction (Cluster 2) was enriched for cell cycle progression, indicative of a proliferative zone. Deeper cortical zones (Clusters 3, 4) showed enrichment for lymphocyte differentiation and T cell maturation. Finally, medullary clusters (Clusters 6, 7) were highly enriched for GO terms related to antigen presentation and MHC protein complex assembly, consistent with the function of APCs（Antigen-Presenting Cells) in negative selection.

Thirdly, to evaluate the biological validity of GATCL-derived spatial representations, we perform PAGA trajectory inference. As depicted in Fig. 5c, the result reveals a clear developmental pathway from the outer (cortical) to the inner (medullary) regions [42]. This is in strong agreement with the established biological process of directed cell migration and differentiation within the mouse thymus. These findings provide compelling evidence that GATCL operating without supervision, effectively clusters cells, preserves their spatial coherence, and successfully identifies spatial domains of high biological significance.

Finally, we leverage the learned attention weights across clusters to investigate GATCL’s internal preferences regarding modality and graph structure. For the RNA modality, shown in Fig. 5, spatial graphs consistently receive higher weights compared with feature graphs across most clusters, particularly in Clusters 0, 3, 4, and 5. In contrast, from Fig. 5e, protein modality demonstrates more balanced reliance on spatial and feature graphs. In addition, according to Fig. 5f, when examining the fusion between RNA and protein modalities, GATCL exhibits strong adaptivity: RNA dominates in Clusters 2–4, whereas protein contributes more in Clusters 0, 1, and 5.

### Application to spatial RNA-ATAC datasets

We extend GATCL to the joint analysis of the spatial transcriptome and spatial epigenome, which is a more challenging field of spatial multi-omics. Specifically, we conduct experiments on a mouse brain dataset downloaded from https://zenodo.org/records/7480069 and a human placental dataset downloaded from https://singlecell.broadinstitute.org/single_cell/study/SCP2601. We follow [43] to preprocess the mouse brain dataset. As for the human placental dataset, apply Latent Semantic Indexing to the original peak count data to reduce its dimensionality to 200. Subsequently, genes expressed in <10 spots are filtered, log-normalized with SCANPY, and the top 3000 HVGs are selected.

**Figure 6:**
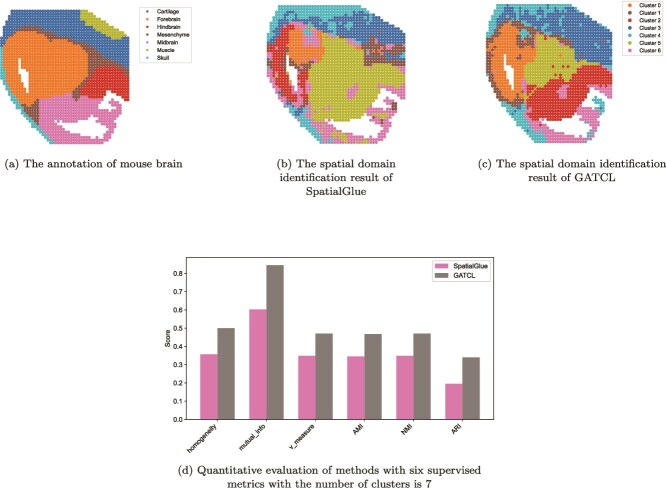
GATCL identifies spatial domains in mouse brain.

**Figure 7:**
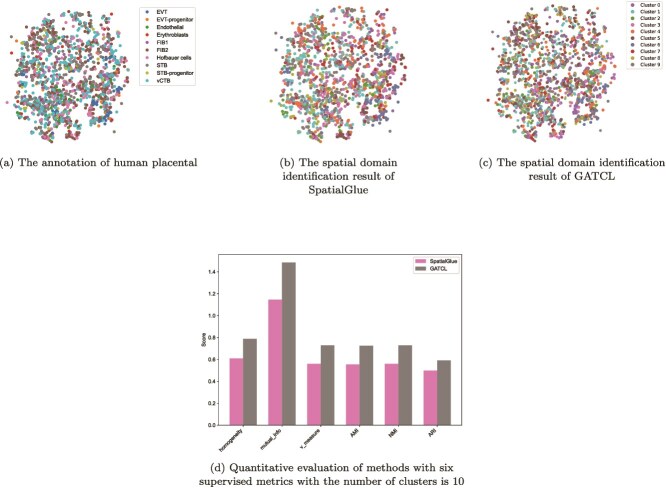
GATCL identifies spatial domains in human placental.

With reference to the ground-truth regional annotation shown in Fig. 6a, by comparison, the spatial domain identification result generated by GATCL (Fig. 6c) exhibits more reasonable regional partitioning than that generated by SpatialGlue (Fig. 6b). GATCL can better capture the overall spatial structure and boundary characteristics of mouse brain regions, while SpatialGlue shows more fragmented and less coherent domain partitioning. For quantitative performance evaluation, we employ six supervised metrics including homogeneity, mutualinfo, Vmeasure, AMI, NMI, and ARI. As shown in Fig. 6d, GATCL consistently achieves higher scores than SpatialGlue across all these metrics. The most significant gains are observed in mutual_info, where GATCL outperforms SpatialGlue by 0.242. Similarly, GATCL achieves 0.144 points higher than SpatialGlue’s about ARI. Substantial advantages are also recorded in homogeneity (+0.143), AMI (+0.123), and v_measure (+0.122), confirming the superior accuracy and robustness of our method. Besides, Fig. 7a shows the ground-truth annotations of human placental, while Fig. 7b and c displays the spatial domain identification results of SpatialGlue and GATCL, respectively. Quantitatively (Fig. 7d), GATCL consistently outperforms SpatialGlue across all six supervised metrics. It is most significant in mutual_info, where GATCL surpasses SpatialGlue by 0.3380 points. And the robust lead is consistent across other key metrics, including homogeneity (+0.1797), AMI (+0.1702), and ARI (+0.0923), highlighting the enhanced robustness of our method. In summary, both qualitative visualization and quantitative evaluation results confirm that GATCL is more effective in identifying spatial domains.

## Conclusion

In this study, we propose GATCL to identify spatial domains more accurately with spatial multi-omics data. GATCL adopts attention-weighted aggregation to selectively prioritize functionally relevant neighbors, thus preserving the precise domain boundaries while CL achieves a robust alignment by bypassing the noise-prone cross-modal reconstruction inherent in existing methods. GATCL has been fully validated on a wide range of datasets, encompassing diverse species, tissue types, and multiple spatial omics such as transcriptomics, proteomics, and chromatin. Complementing the direct performance comparison, the ablation analysis confirms both the graph attention mechanism and cross-modal CL make substantial contributions to precise spatial domain depiction. Furthermore, the parameter sensitivity analysis is also conducted (Supplementary Materials) to assess the impact of hyperparameter choices on model performance. Finally, both training and inference processes are efficient, and can be completed in a few tens of seconds on an Intel(R) Xeon(R) Silver 4316 CPU and NVIDIA A40 GPU.

By reliably identifying biologically meaningful spatial domains, GATCL might reveal tumor microenvironment interactions, providing critical insights for targeted therapeutic strategies. Looking ahead, we aim to extend the GATCL framework to further incorporate Hematoxylin and Eosin (H&E)-stained tissue images, thereby unlocking deeper insights into the interplay between spatial architecture and molecular expression.

**Key Points**

- We propose GATCL which is based on graph attention network and contrastive learning (CL) for spatial domain identification.
- GATCL leverages a graph attention network to overcome the limitations of static graph models, dynamically weighing cellular neighbors to achieve precise delineation of functional domains.
- A CL framework is employed to align multi-modal representations, bypassing noise-prone cross-modal reconstruction and learning a robust latent space for accurate domain identification.
- Experimental results across six datasets, including spatial transcriptomics, proteomics, and chromatin from different platforms, demonstrate that GATCL outperforms seven representative methods across six evaluation metrics.

## Supplementary Material

Supplementary_Material_bbag043

## Data Availability

The source code is available at https://github.com/1999mjc/GATCL.
