# Supplementary material for: GATCL: graph attention network meets contrastive learning for spatial domain identification
Source: Brief Bioinform. 2026 Feb 12;27(1):bbag043. doi: 10.1093/bib/bbag043 (PMC12900075; doi:10.1093/bib/bbag043)
Supplement: Supplementary_Material_bbag043 [file supplementary_material_bbag043.docx]

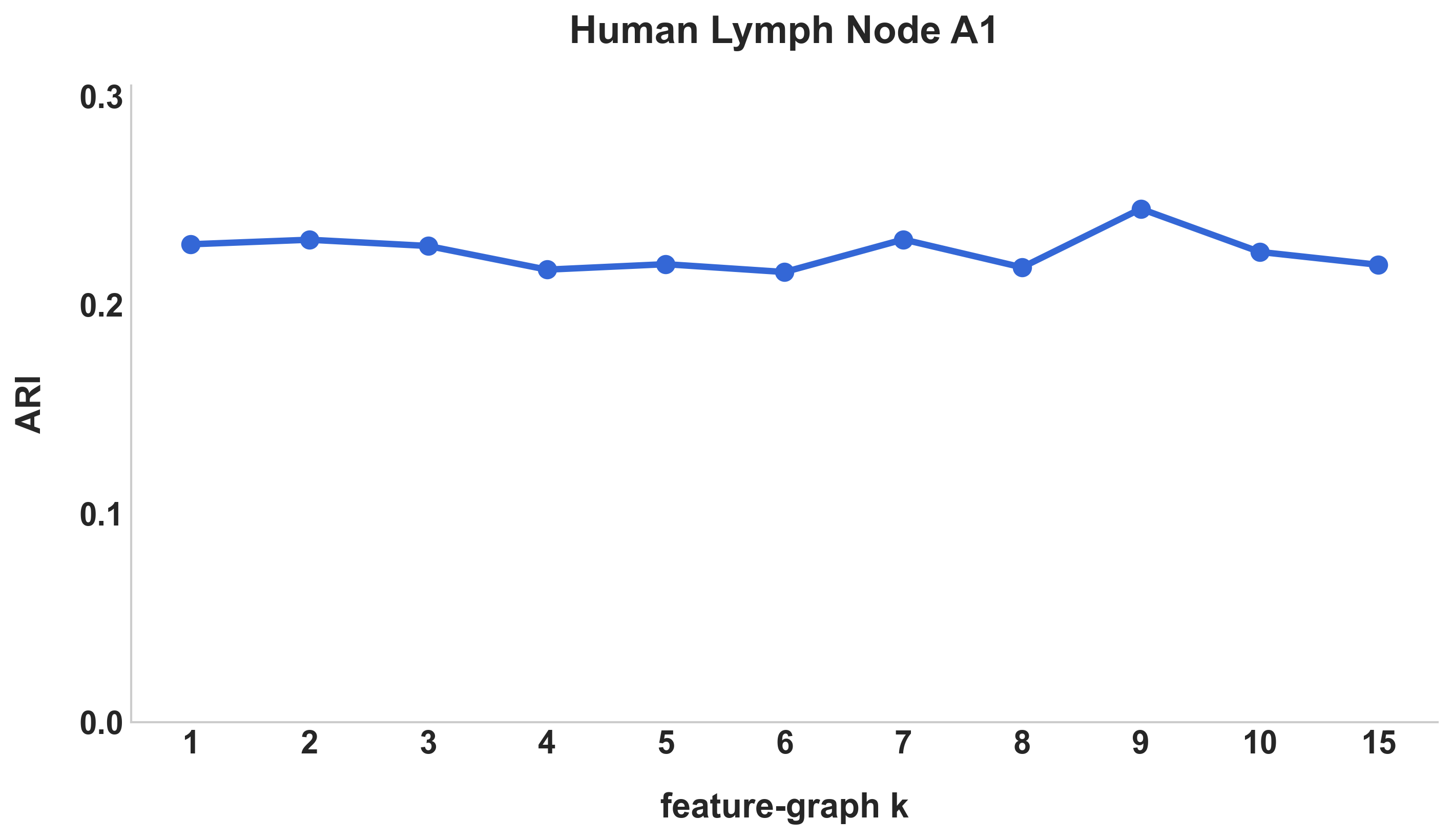

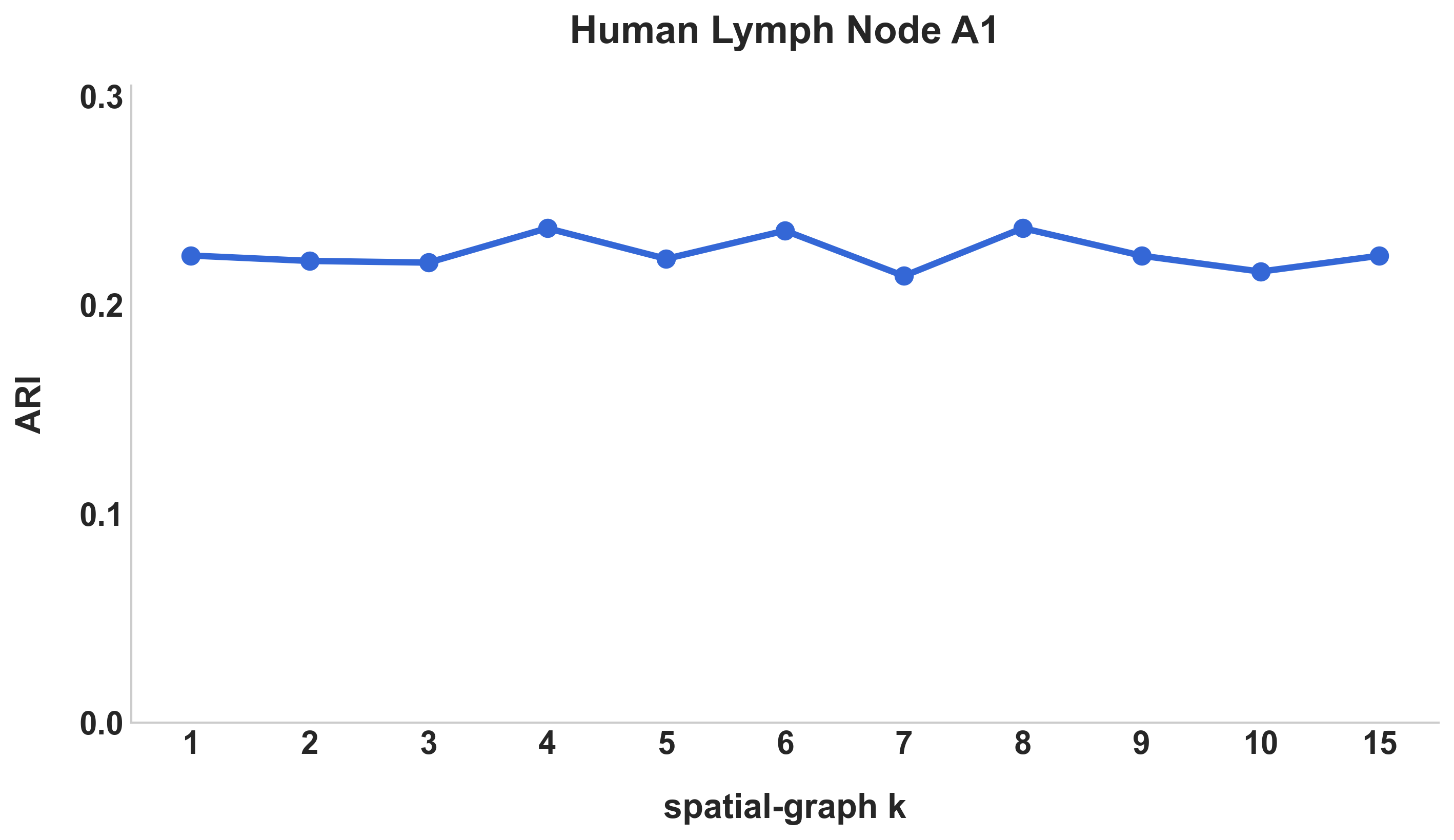


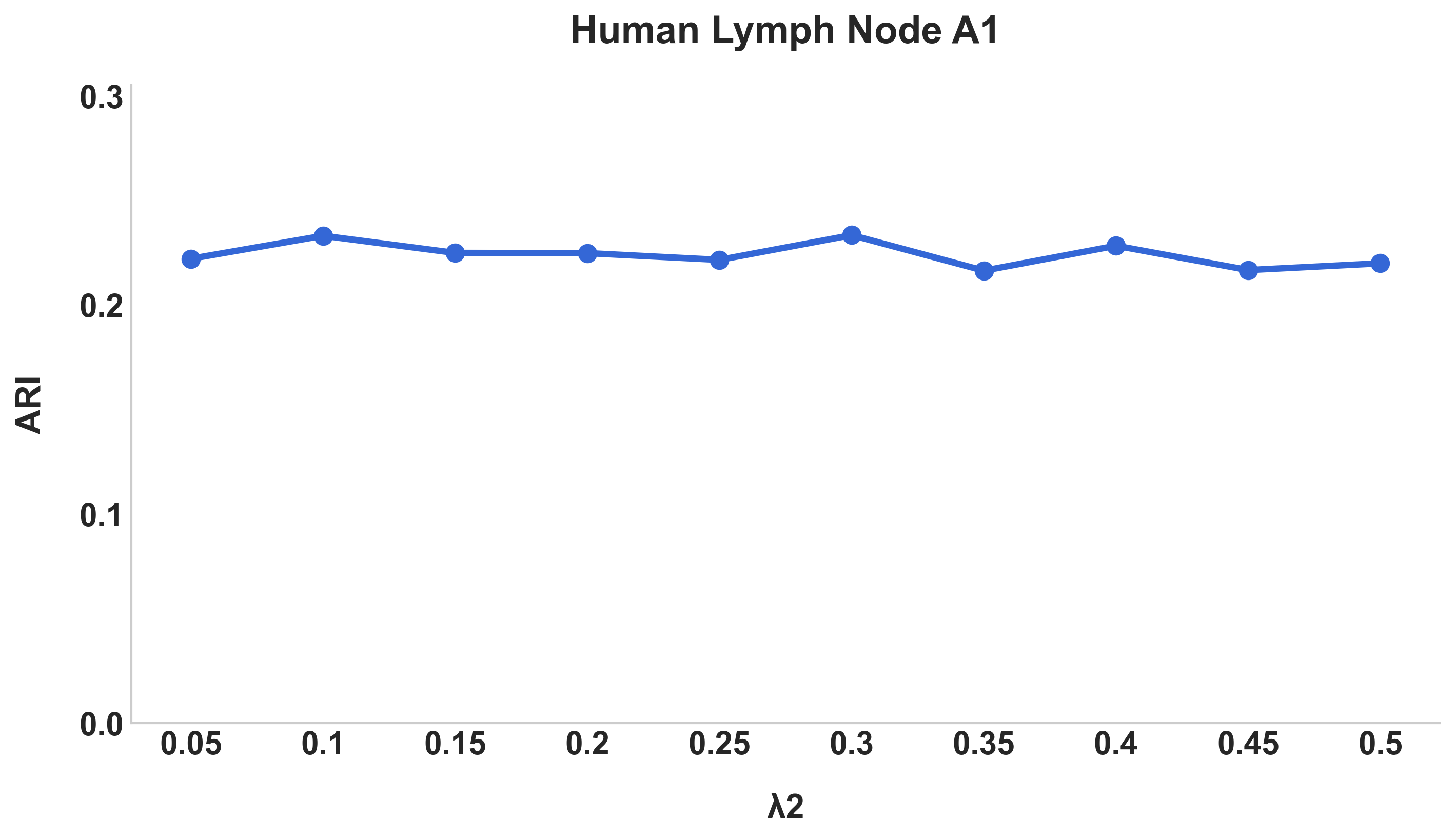
(a) (b)


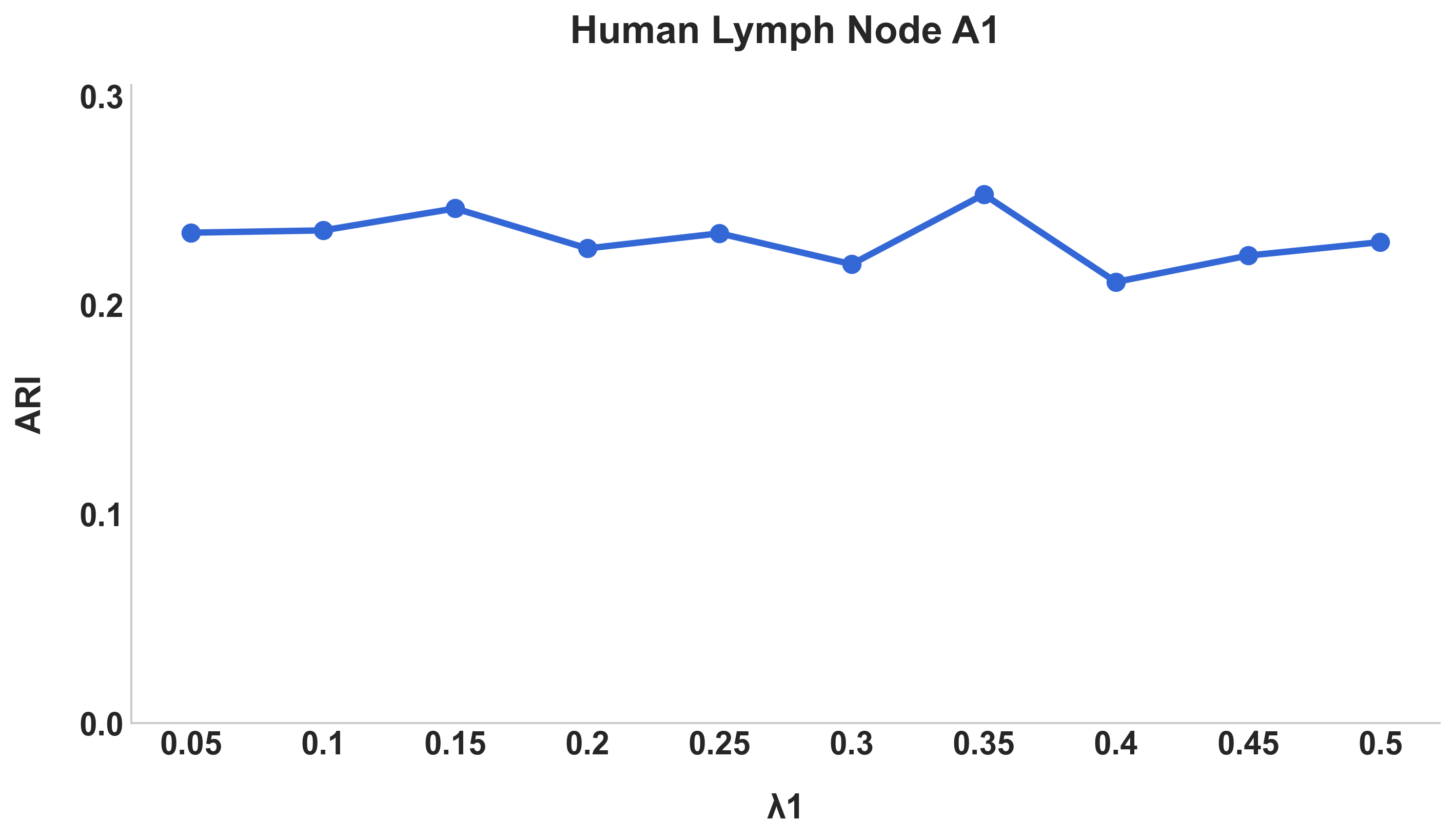


(c) (d)


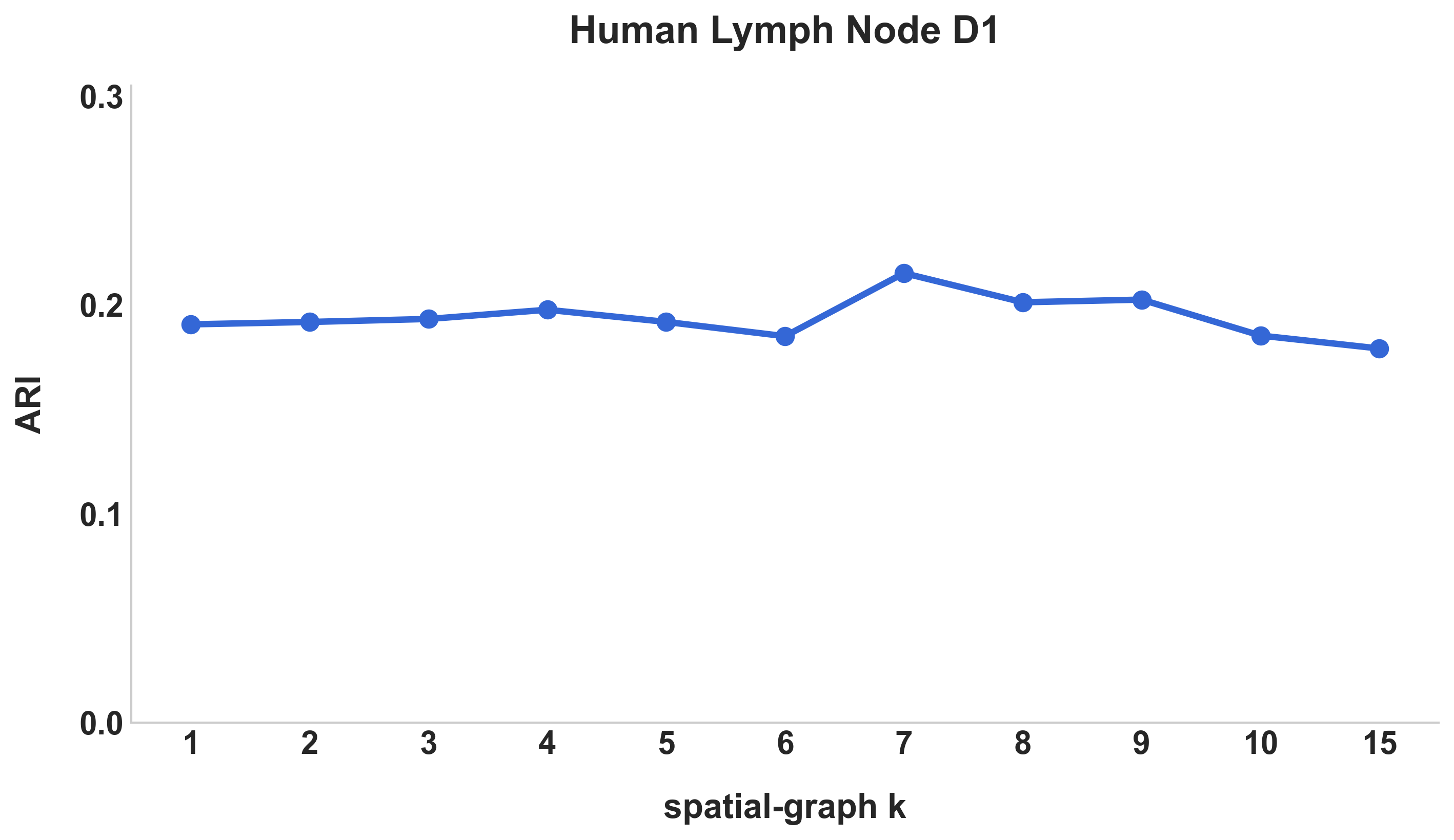

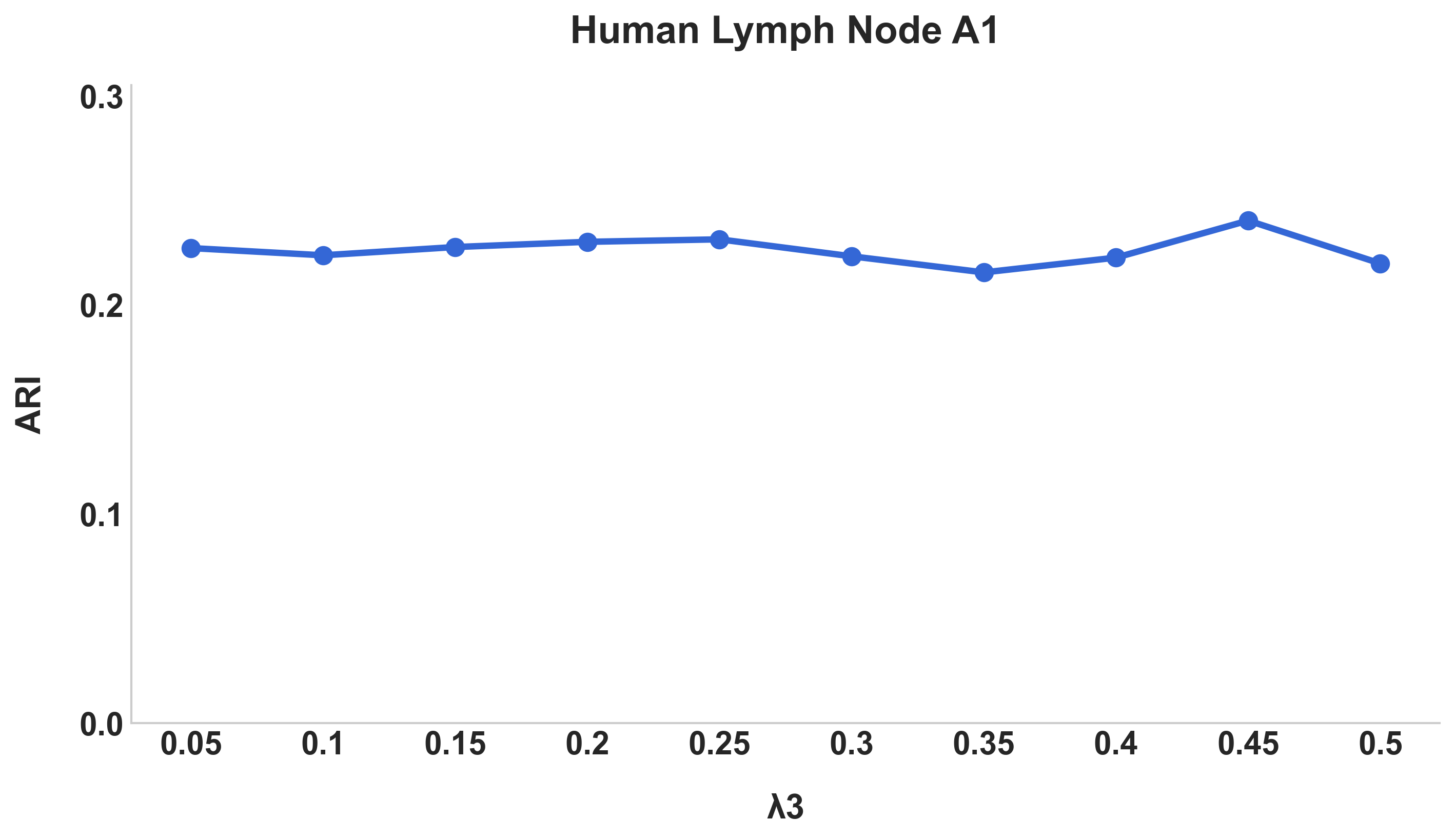


(e) (f)


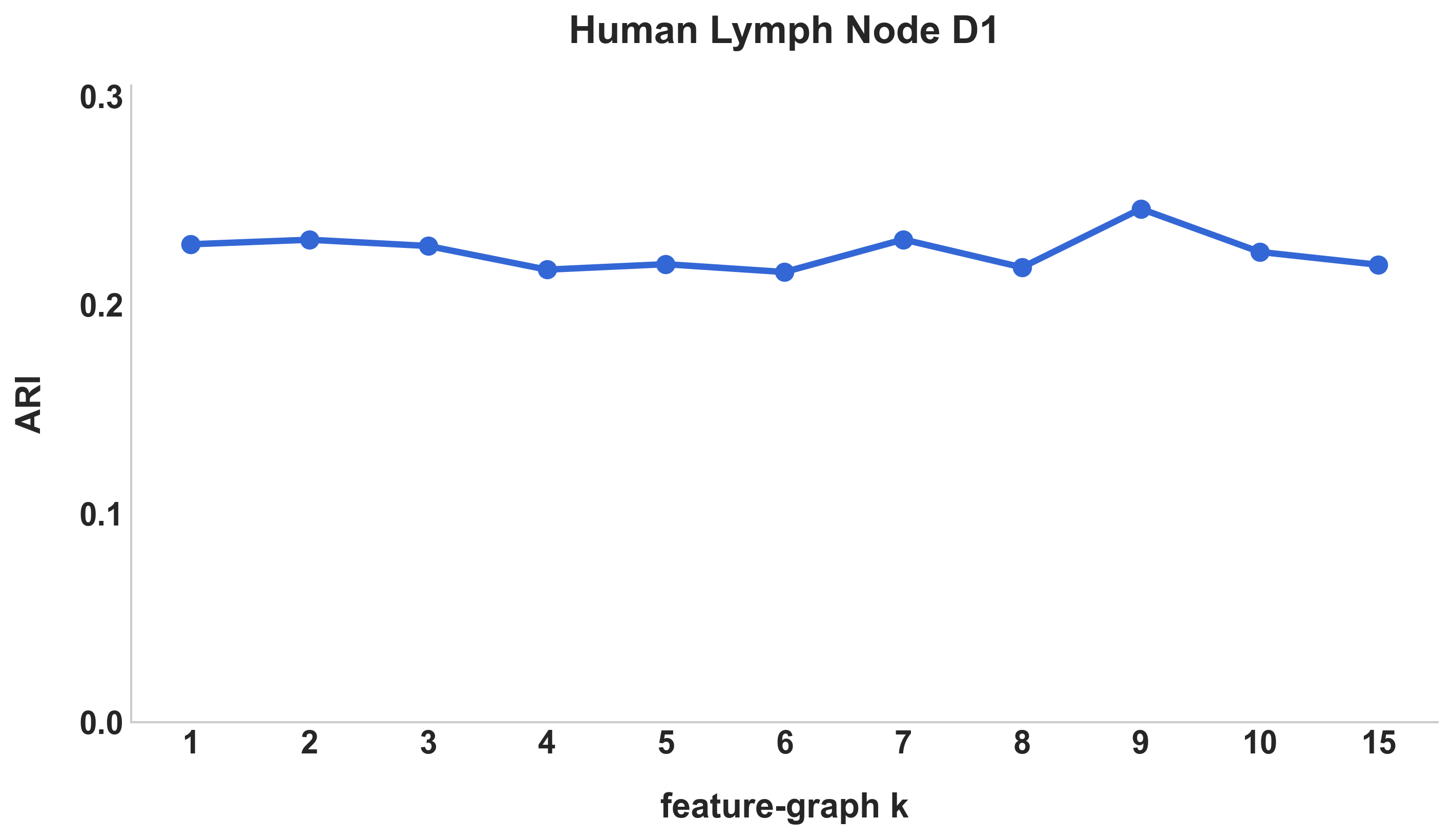

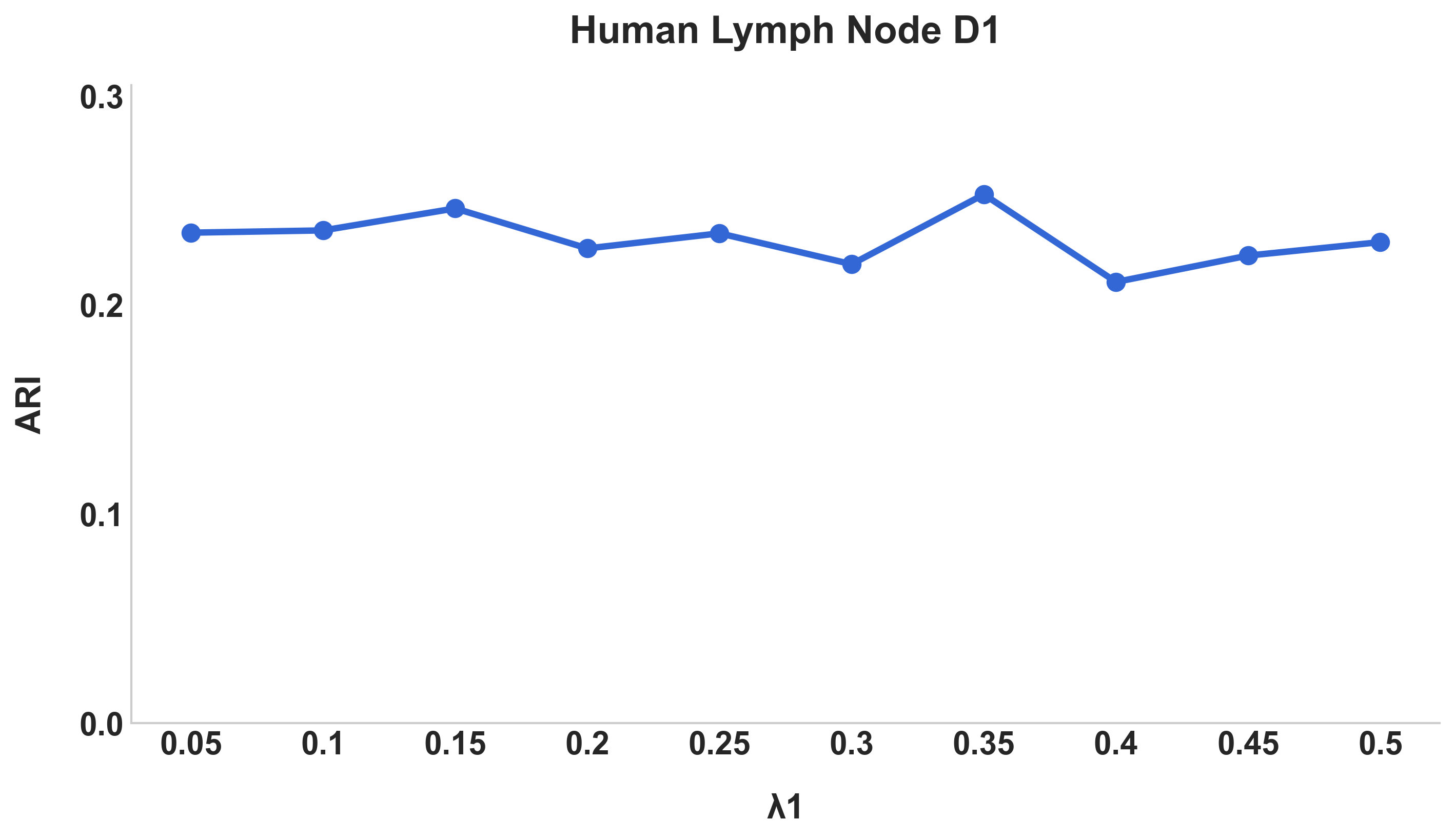


(g) (h)


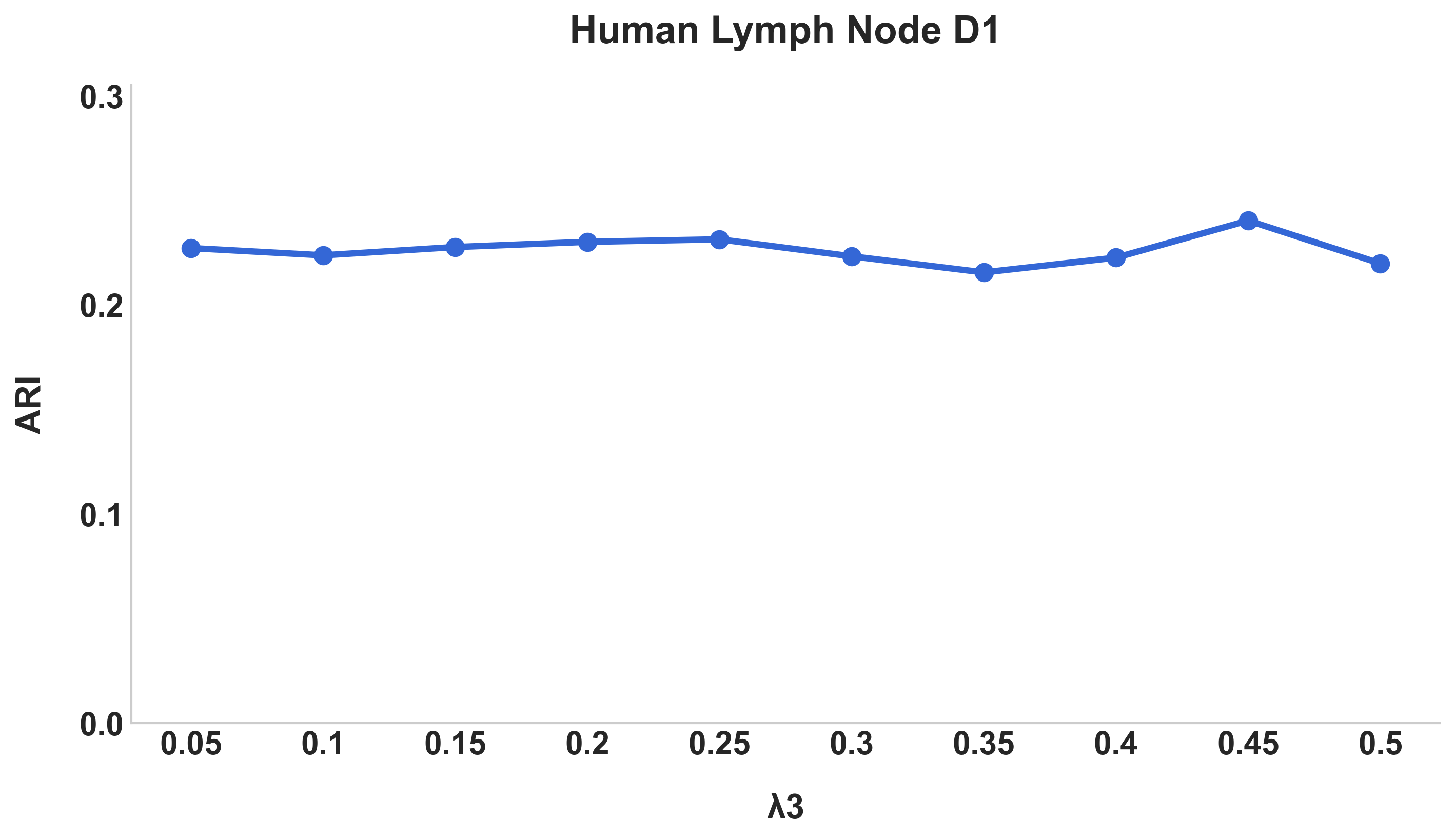

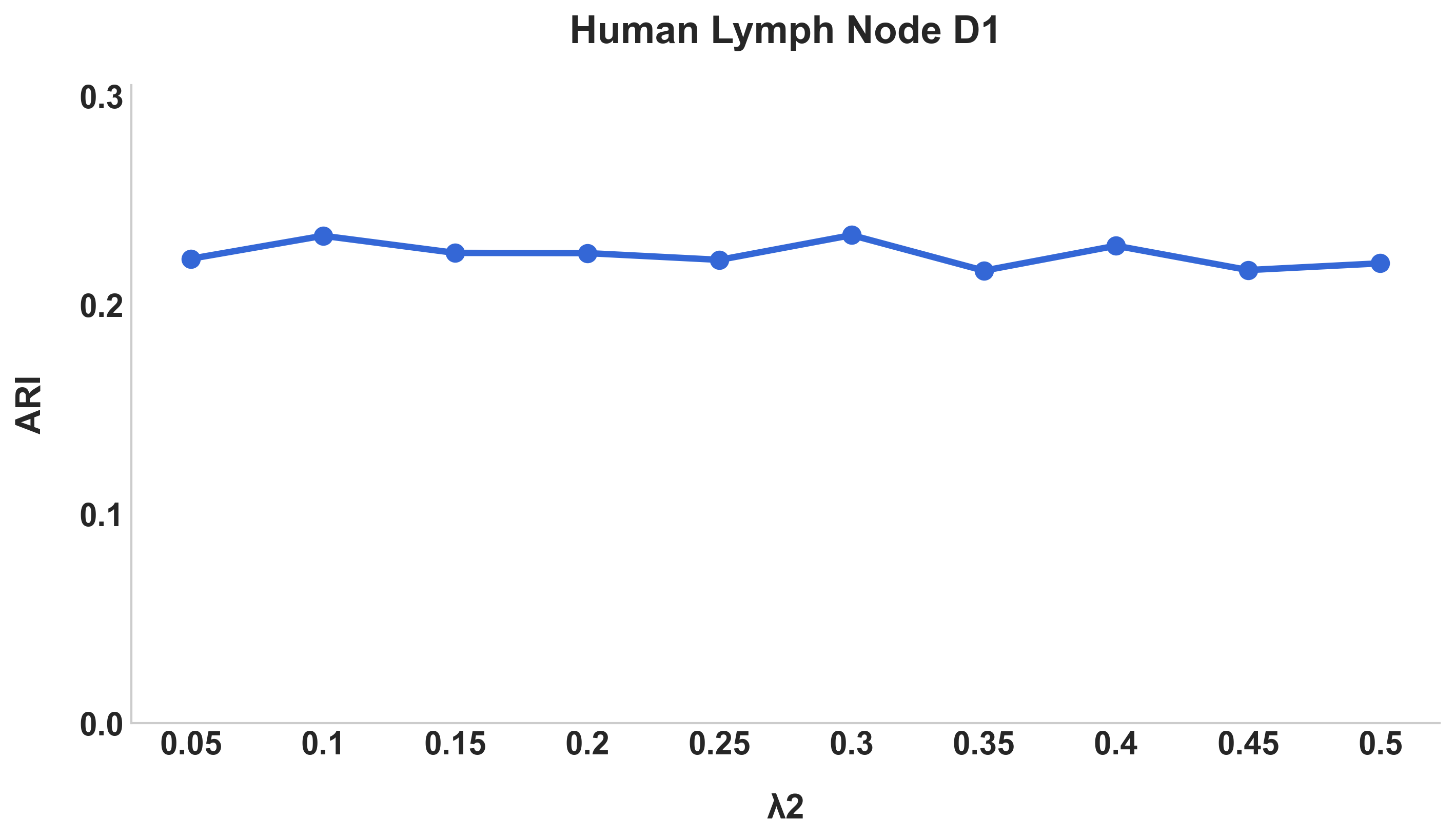


(i) (j)


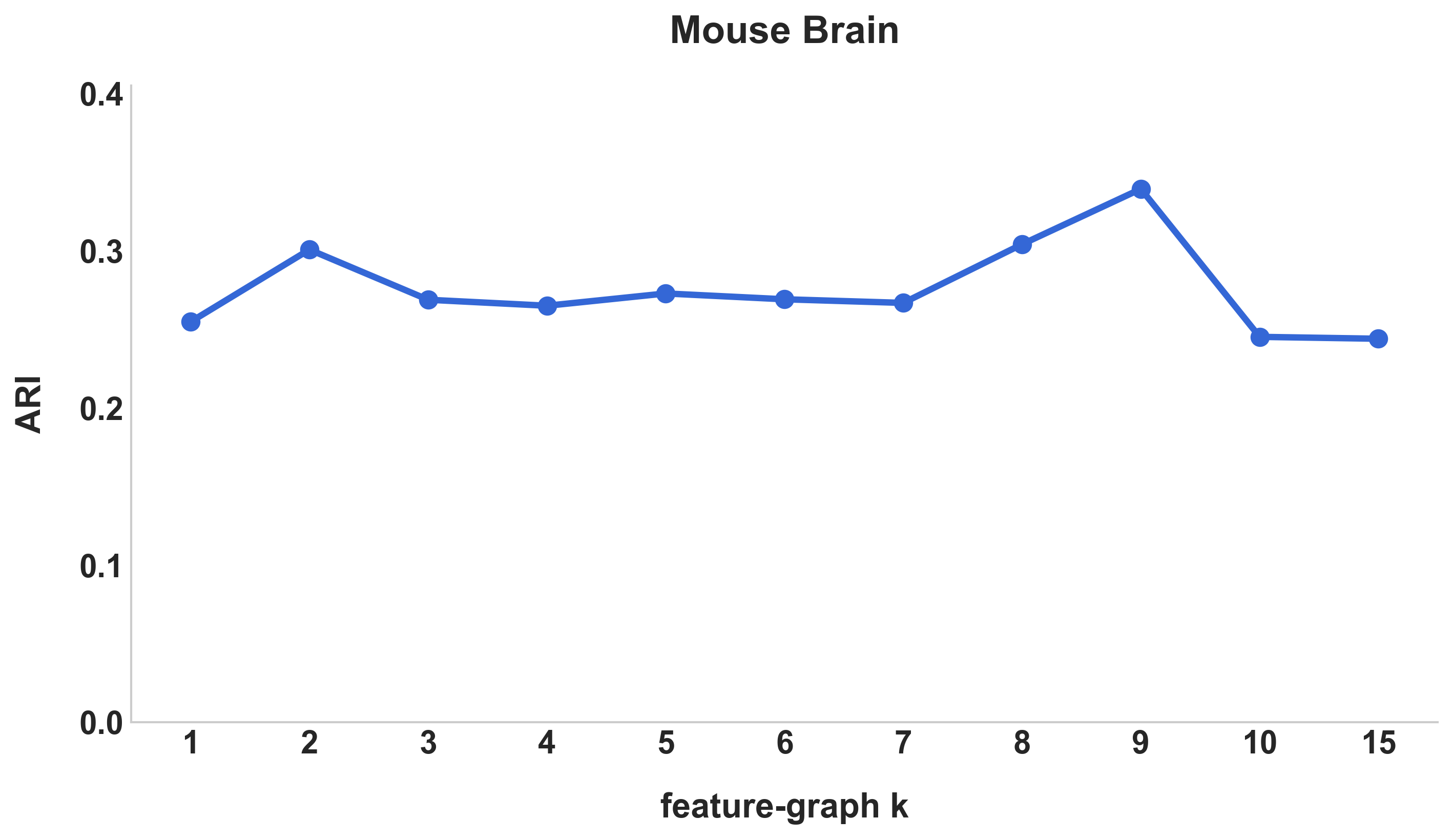

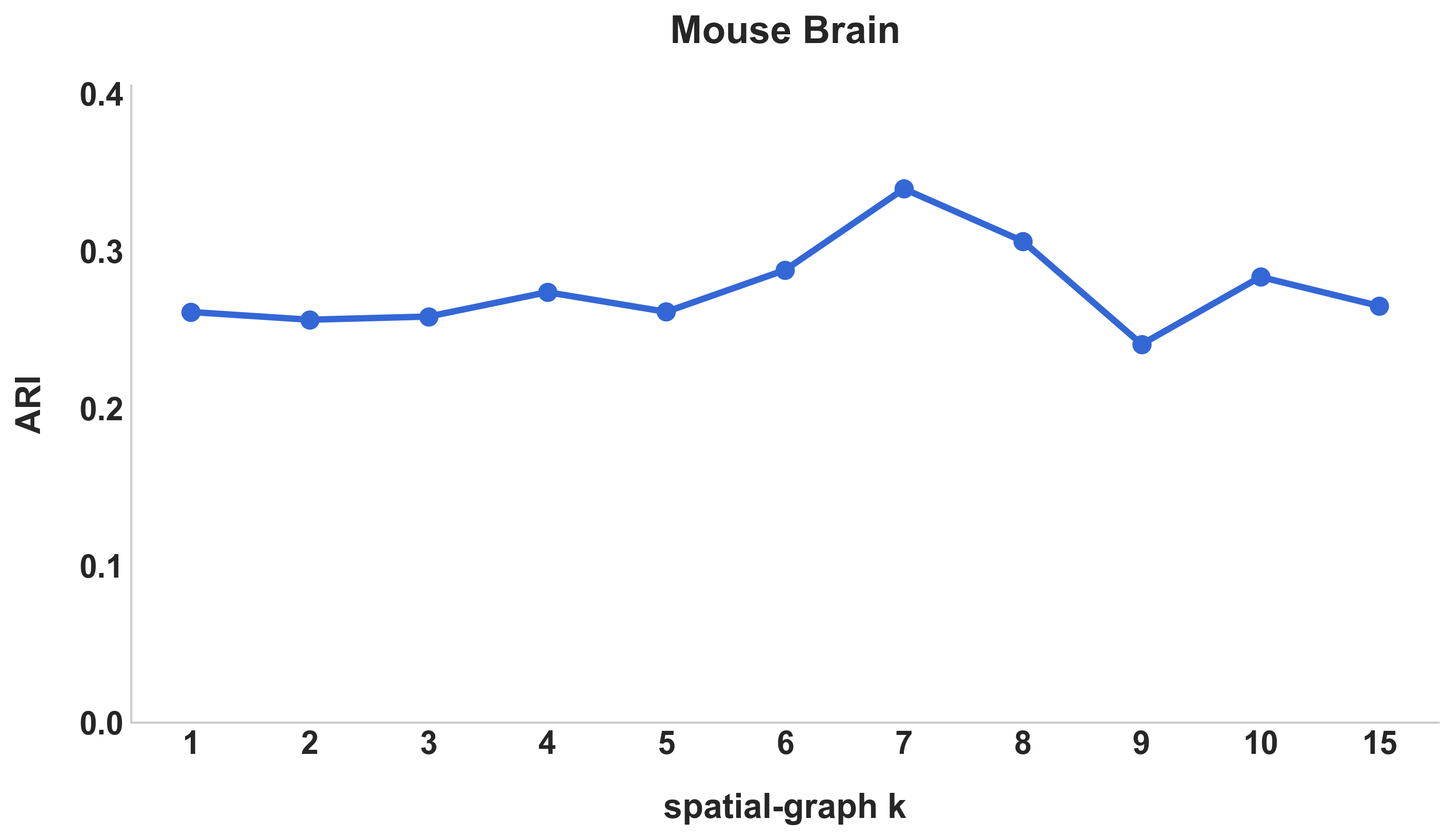


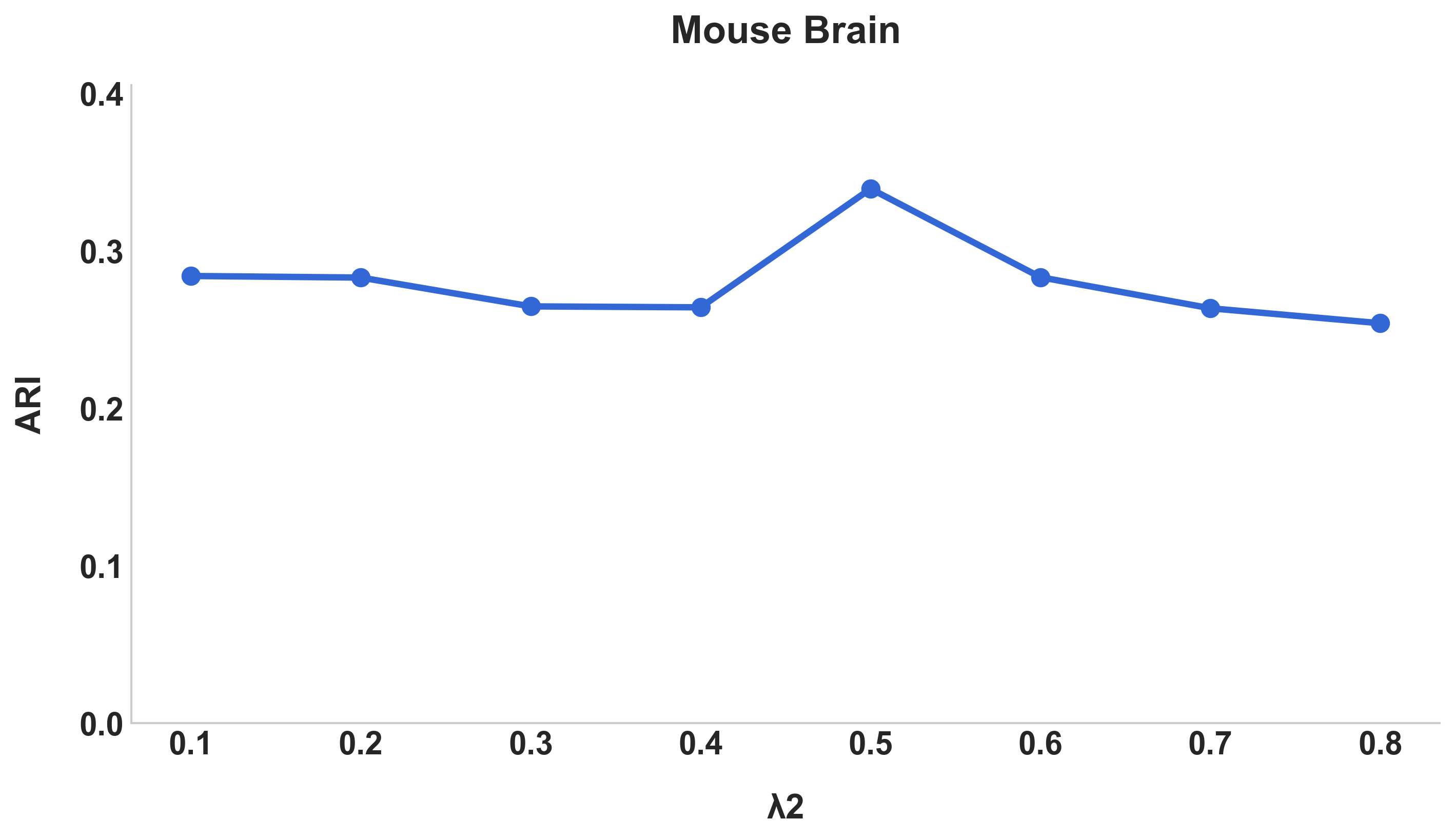
 (k) (l)
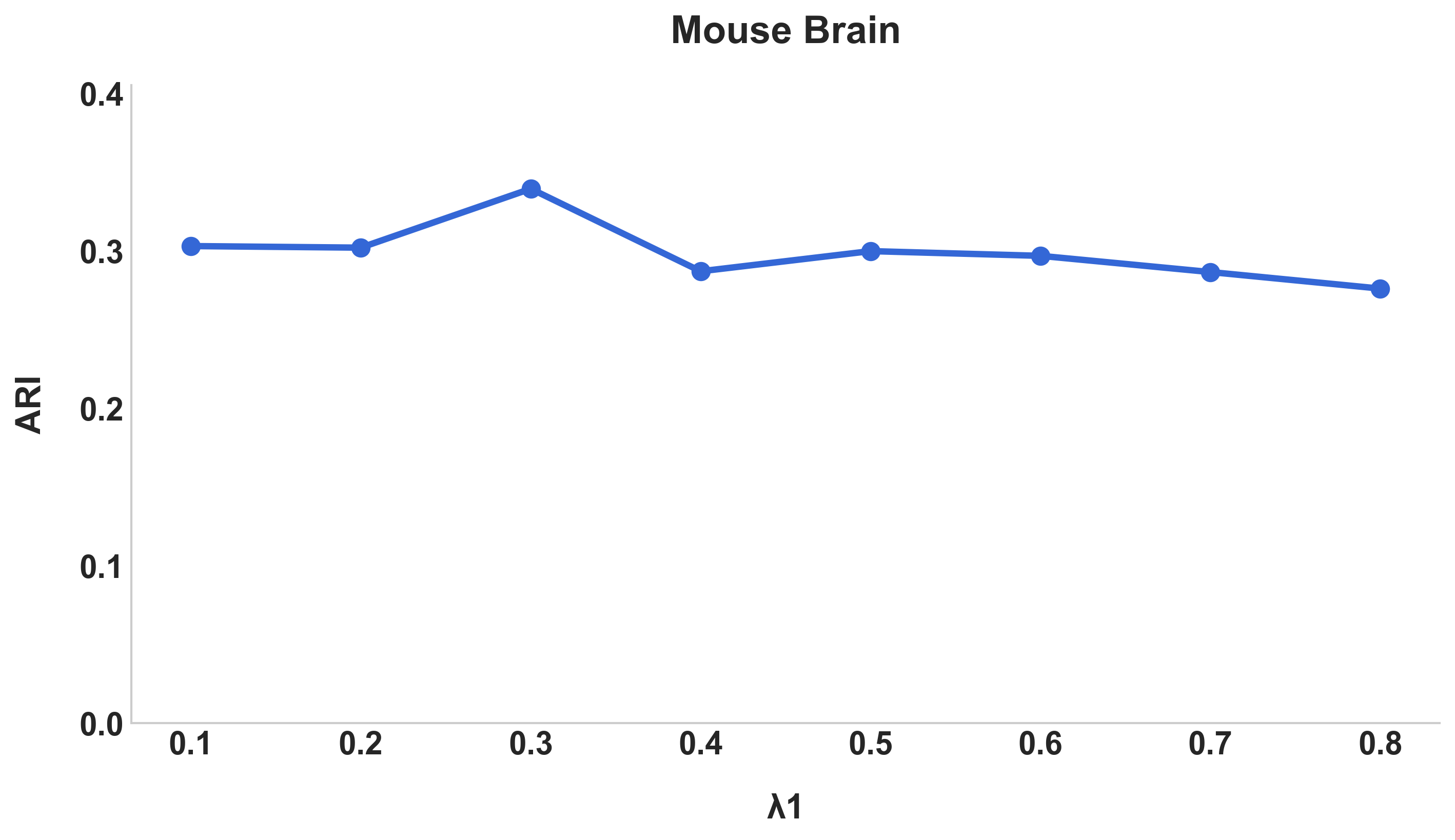


(m) (n)


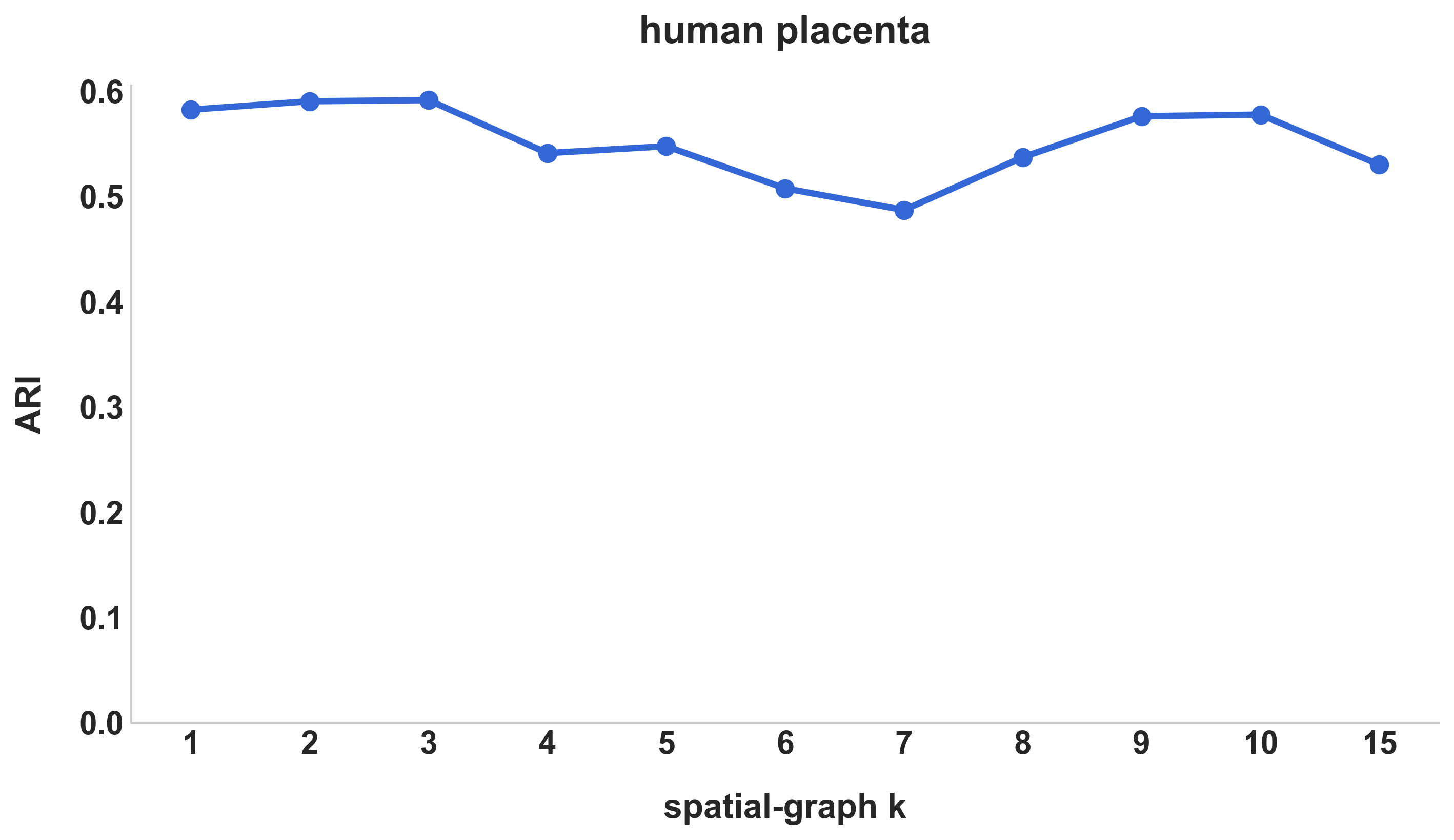

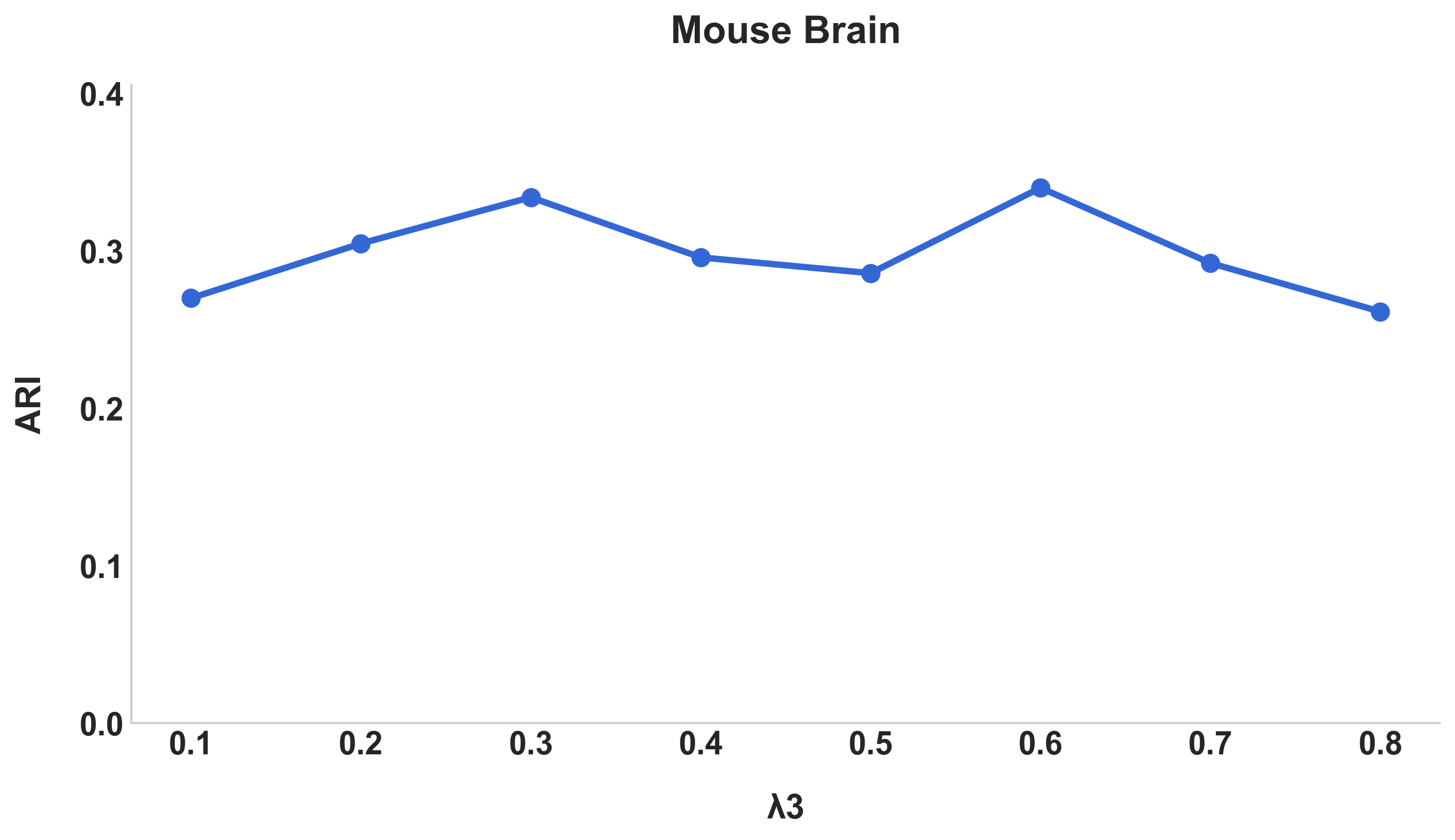


(o) (p)


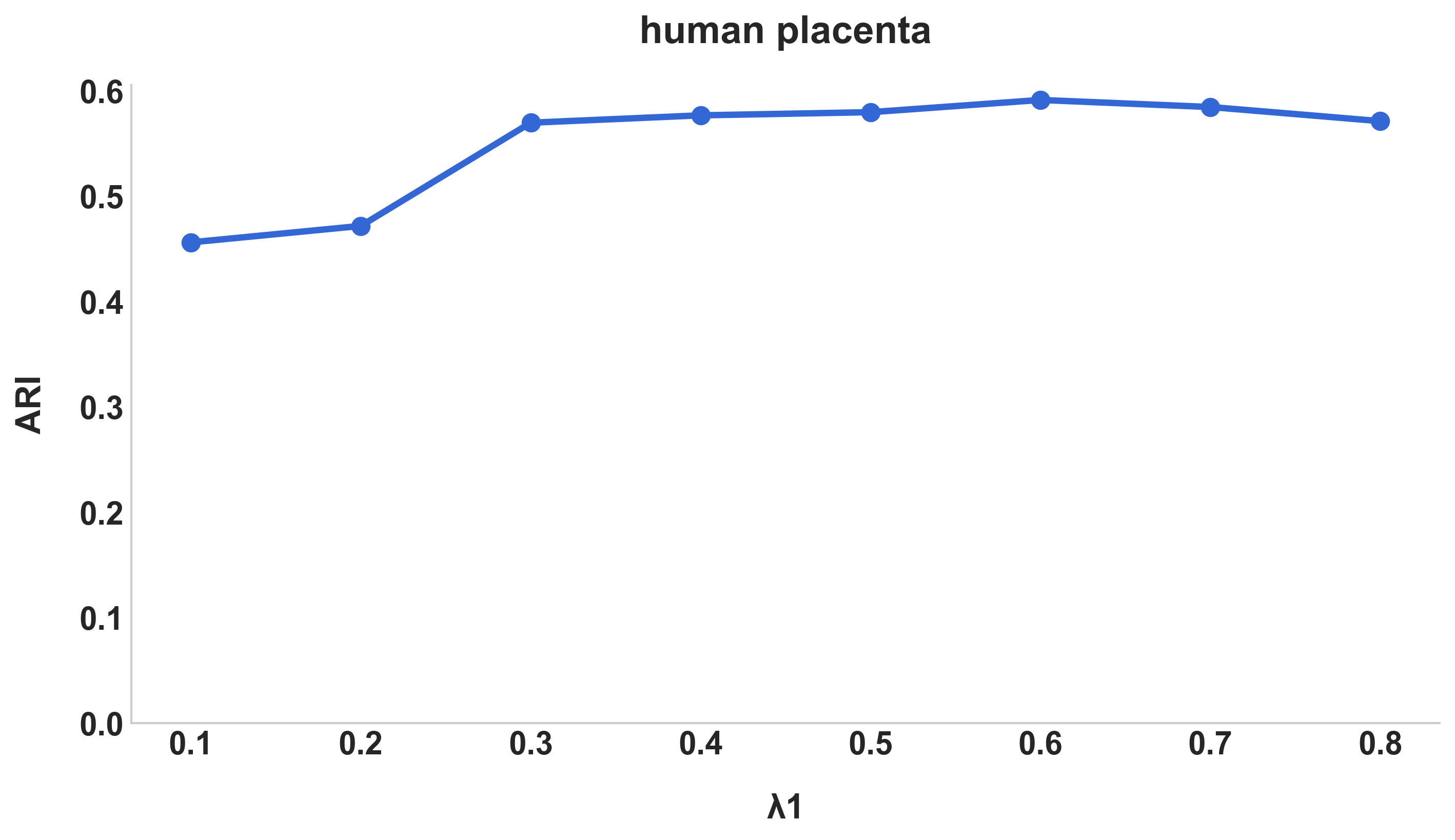

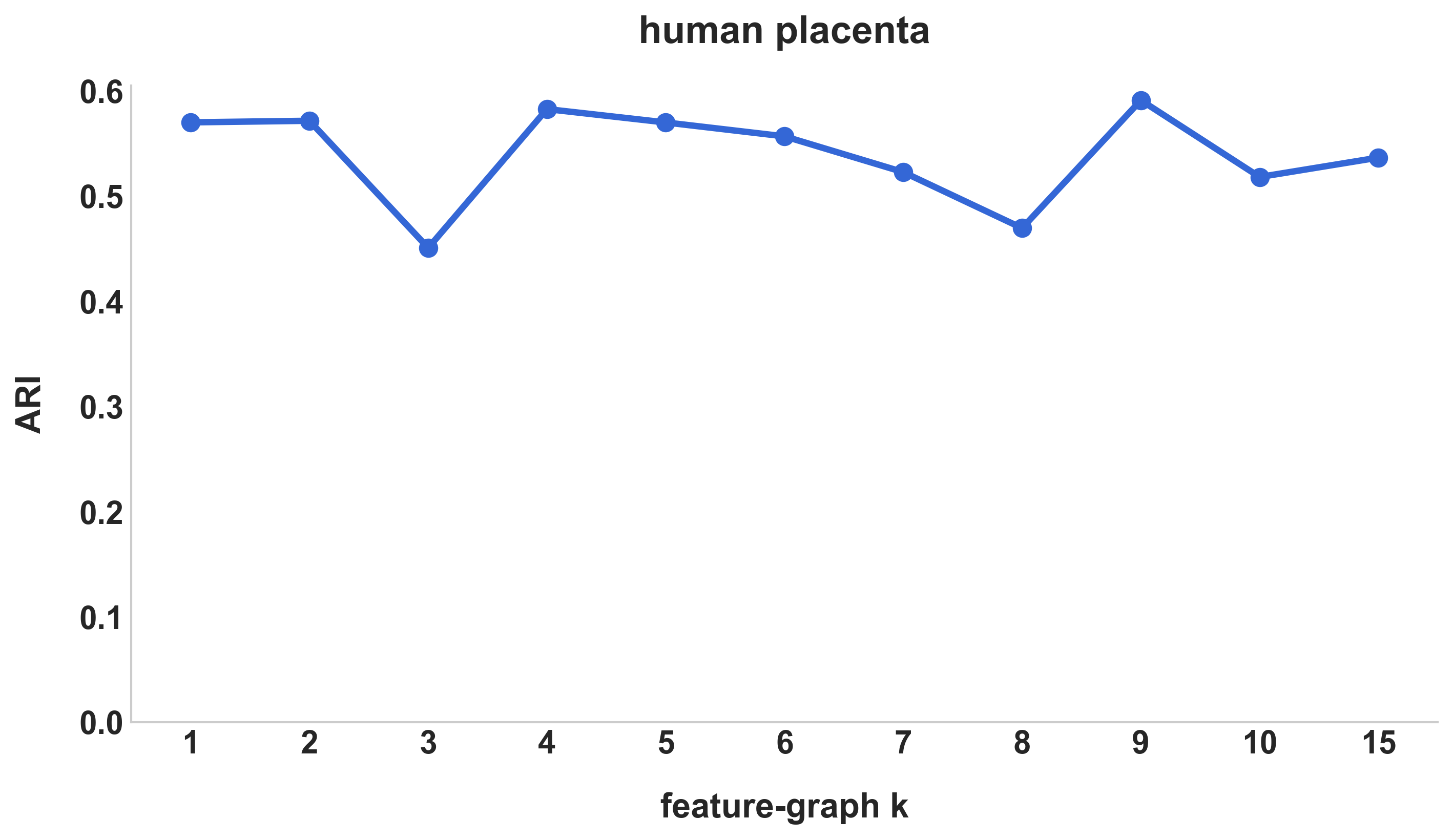


(q) (r)


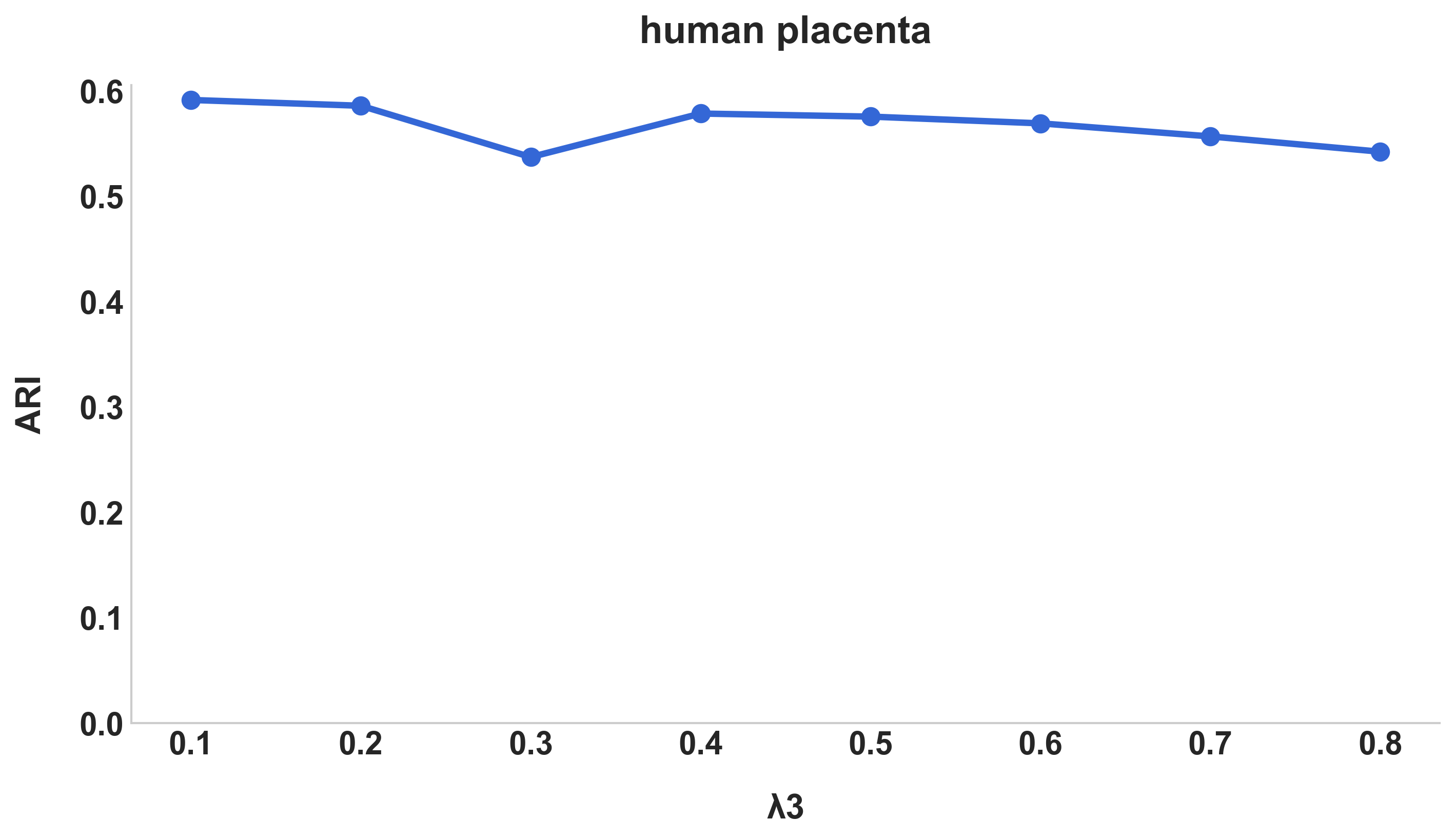

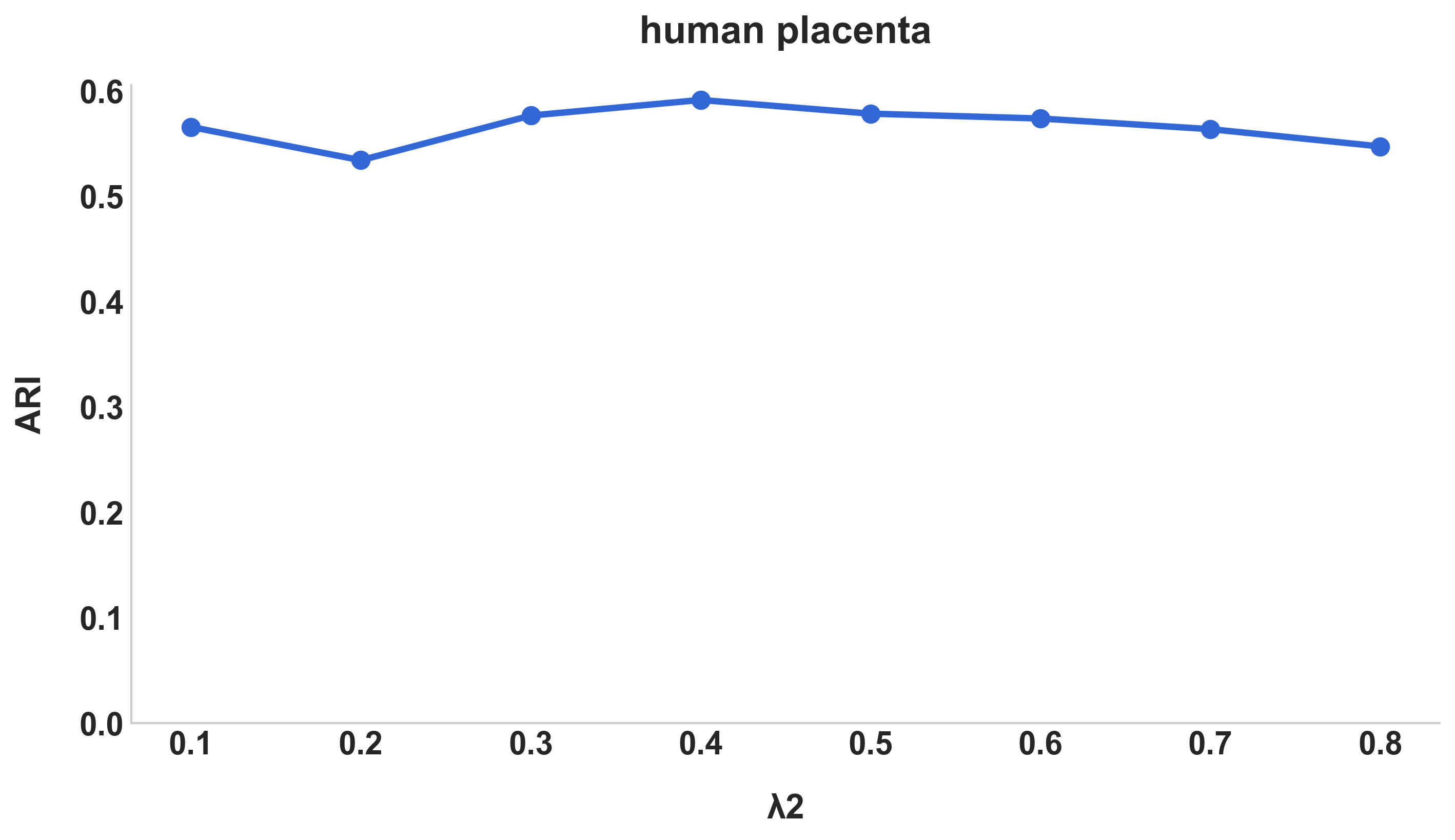


(s) (t)

Supplementary Figure 1. Hyperparameter sensitivity analysis. (a)-(e) Sensitivity analysis on human lymph A1. (f)-(j) Sensitivity analysis on human lymph D1. (k)-(o) Sensitivity analysis on mouse brain. (p)-(t) Sensitivity analysis on human placenta.
